# In the belly of the beast: How dietary changes and preexisting invasive prey may have promoted the success of a novel invasive amphibian

**DOI:** 10.1371/journal.pone.0352970

**Published:** 2026-07-10

**Authors:** Maya J. Williams, Julia L. Riley, James Baxter-Gilbert

**Affiliations:** Department of Biology, Mount Allison University, Sackville, New Brunswick, Canada; IEAPM: Instituto de Estudos do Mar Almirante Paulo Moreira, BRAZIL

## Abstract

Biological invasions provide unique opportunities to examine how populations cope with rapid ecological change, as well as the influence of human activity on our natural world. The island of Newfoundland, Canada, has no native amphibians; however, five species have established non-native populations, including one caudatan, the Eastern Red-backed Salamander (*Plethodon cinereus*). We posit that this salamander’s successful colonisation may have been mediated by dietary shifts that allowed them to capitalize on a greater variety of prey, as well as by exploiting pre-existing invasive prey communities – both these mechanisms could limit the likelihood of competitive exclusion and promote establishment in a novel environment. To examine this, we identified stomach contents of Newfoundland Eastern Red-backed salamanders, characterised the prey as native or invasive, and compared the dietary composition of the invasive population to that of conspecifics in the native range through a systematic literature review. As predicted, the invasive salamander population’s diet is more generalised (i.e., a broader dietary niche) than the native salamanders, and invasive invertebrates comprised two thirds of the volume of prey eaten by invasive salamanders. Our research provides insight into the Niche Breadth Invasion Hypothesis as well as the Invasional Meltdown Hypothesis, which are mechanisms that may have allowed this species to establish a population in an island ecosystem. Furthermore, our research suggests that prior invasions of a diverse invertebrate community to Newfoundland may have bolstered the invasive potential of this novel, non-native salamander.

## Introduction

Invasive species are a globally pervasive threat to biodiversity, whose successful establishment and subsequent spread are often directly or indirectly mediated by human activity and the biological flexibility of the invader [1–3]. As agents of ecological change, invasive populations can damage biological communities by disrupting trophic relationships, nutrient cycling, and resource availability for native species [2]. For example, invasive House Cats (*Felis domesticus*) and rats (*Rattus* spp.) are leading causes of biodiversity declines and extinctions on islands across the globe due to their predatory nature [4,5]. Meanwhile, Cane Toads (*Rhinella marina*) native to South America, have been introduced to over 40 countries [6] and are known to have a wide range of impacts through ecological disruption [7,8], including competition at lower trophic levels [9], and severely diminishing apex predator populations leading to widespread, detrimental trophic cascades [10]. These examples highlight the ecological threat invasive populations can pose. In addition, there can be significant socio-economic costs associated with managing and mitigating threats [11]. However, not all invasive populations innately cause negative ecological or economic impacts. Some invasive populations have been viewed as neutral or even potentially beneficial within certain contexts [12]. Nevertheless, the potential for negative impacts does exist and the uncertainty around this is greater for novel taxa establishing an invasive population for the first time. Despite increasing efforts to curtail their spread, the introduction and establishment of new invasive populations across the globe continues to rise annually [13]. This continued increase in the number of invasive taxa is largely due to growing anthropogenic activity, such as expanding transportation networks and increased habitat alteration [13].

Although the rates of new species introductions continue to steadily increase [13], the outcome for most invasions is failure – owing to the many abiotic and biotic barriers encountered during the process [14,15]. Invasive populations can form after non-native individuals progress through a series of ‘invasion stages’, whereby they must be able to survive transport to a new location, survive within the new habitat, reproduce and maintain a self-sustaining population, and expand their range beyond the site of initial establishment [14,16]. Between each of these invasion stages are ecological barriers, such as an inability to survive transport or successfully breed within the novel habitat, that can lead to invasion failure [14]. In fact, it is expected that, due to these barriers, most potential biological invasions fail, especially after accidental introductions [15]. For example, although the Cane Toad is one of the most significant vertebrate invaders of the last century [8,10], their introductions into Mauritius and Tanzania both failed [17]. As such, the drivers and mechanisms that enable non-native populations to overcome the barriers between invasion stages and, in turn, allow an invasion to be successful, has garnered significant attention in the form of a series of ‘invasion success hypotheses’ [3]. These hypotheses encompass a variety of factors that are associated with enhancing invasive potential, spanning biological, ecological, and evolutionary influences [3]. For example, some invasions are thought to have been successful because the species had a high degree of phenotypic plasticity (e.g., Plasticity Hypothesis [18]), or they are no longer suppressed by their evolved predators which are absent in the invaded ecosystem (e.g., Enemy Release Hypothesis [19]), or that the species possess certain traits, like defences, that provide them enhanced survival in comparison to native taxa (e.g., Novel Weapons Hypothesis [20]). Another branch of these invasion hypotheses, however, pertains to the fundamentals of whether a non-native population is capable of integrating into a novel niche space, such as acquiring enough resources to sustain a viable population [3].

One of the leading means by which invasive animal populations can become established and spread outward from their introduction site is their ability to capitalise on novel food resources and/or leverage a generalist diet [2,21,22]. A broad ecological niche, including flexible diet and habitat use, has been seen to support many species’ successful invasions [21]. The value of a broad dietary niche promoting invasion success is linked to the concept that if a colonizing population can more widely diversify what they eat, then they can minimise the degree of direct competition with specific native taxa and thus increasing their invasive potential (i.e., Niche Breadth Invasion Success Hypothesis [23]). For example, the African Clawed Frog (*Xenopus laevis*) is a globally invasive amphibian with a generalist diet [24]. However, during its invasion in France it expanded its dietary niche breadth compared to native populations in South Africa [25]. Similarly, when the dietary niche breadths of invasive populations of the Round Goby (*Neogobius melanostomus*) and Tubenose Goby (*Proterorhinus semilunaris*) were compared, the more successful invader within the Laurentian Great Lakes (i.e., Round Goby) had a broader and more plastic diet [26]. Although expanding a species’ dietary options or preferences may enhance some populations’ abilities to invade, this trend is not seen across all invasions [27] and can even be antithetical (i.e., dietary niche constriction; see [28]). Regardless, dietary plasticity may not be sufficient to allow the establishment of a potential invasive population if most of the evolved niches are filled [29]. To overcome this challenge, invading populations may require some form of disturbance that disrupts an ecosystem’s *status quo* to create resource opportunities [29–31] or for novel opportunities to be created by the presence of pre-existing invasive taxa [31].

If a given niche is being fully exploited, the potential for an introduced population to establish may be diminished, as native species may possess a competitive advantage – thus factors that disrupt this equilibrium can better an invasion’s odds. The Invasional Meltdown Hypothesis posits that the success of pre-existing invasive species can facilitate the establishment and proliferation of novel introduced organisms within a given ecosystem [32,33]. Such invasive population interactions can result in the modification of food webs through creation of niche opportunities or provision of underutilised resources that a recent introduced population may capitalise upon to overcome fundamental barriers to establishment [1,14,21]. For example, a global review about invasive American Bullfrog (*Lithobates catesbeianus*) populations found that invasive crayfish are a common and dominant prey group in their diet, with most established invasive bullfrog populations co-occurring with invasive crayfish 76% of the time [34]. The invasion of Crazy Yellow Ants (*Anoplolepis gracilipes*) to Christmas Island caused the decline of multiple species of native land crabs through predation, which opened niche space for a secondary invasion by the Giant African Land Snail (*Lissachatina fulica*) [33,35]. Similarly, fowling mussels (*Dreissena* spp.) in the Laurentian Great Lakes hardened the soft-sediment habitat, thereby promoting the invasion of Ponto-Caspian Amphipod (*Echinogammarus ischnus*) into areas that were previously unsuitable [36,37]. These scenarios, which support the Invasional Meltdown Hypothesis [32], all demonstrate how prior invasions create future invasion opportunities, which provides an additional rationale for why rates of non-native species introductions continue to increase (i.e., a potential positive feedback loop) [13].

As countries endeavour to better understand and manage their invasive species, particular attention should be paid to regions that represent invasive hotspots (i.e., areas with burgeoning invasive communities [38,39]). For example, the island of Newfoundland hosts one of the densest assemblages of domestic invasive species in Canada (i.e., taxa that are native to areas elsewhere in a country, but not to the region of interest [40]). This may be best underscored by the island’s herpetofaunal community, which is entirely invasive, as it has no native amphibians or reptiles. Since European colonization, five species of amphibian have been introduced [40,41]. The most recent addition to this community came from the description of Eastern Red-backed Salamanders (*Plethodon cinereus*) maintaining a self-sustaining population near Conception Bay South, which is the first established caudatan described to the island [42]. To date, little is known about the invasion history or ecology of this extralimital salamander population, however in their native range this species is known to be a generalist predator that will feed on invasive prey when ecological conditions are favorable [43] – suggesting that dietary flexibility may be a trait that could promote invasion success.

Herein, we examine the diet of the invasive population of Eastern Red-backed Salamanders in Newfoundland, Canada, and use this information to test whether this successful invasion is associated with a broadening of this species’ niche breadth (i.e., the Niche Breadth Invasion Success Hypothesis [23]) and/or the use of pre-existing invasive/non-native invertebrate communities to fulfill their resource demands (i.e., the Invasional Meltdown Hypothesis [32]). First, to test the Niche Breadth Invasion Success Hypothesis [23], we predict that the diet of the invasive population of salamanders will have a higher species richness (i.e., number of prey types) and have a more diverse (i.e., larger Shannon Weiner diversity index value) and even (i.e., a larger Pielou’s J score) assemblage compared to that of native populations. To investigate this, we analyzed the stomach contents of salamanders collected from Newfoundland and compared these results to a dataset of this species’ diet across its native range that we generated from a systematic literature review. Second, to test the Invasional Meltdown Hypothesis [32], we predict that the invasive salamander population’s diet will consists of a high proportion of invasive/non-native invertebrates in abundance (> 30% by count) and in biomass (> 30% by volume). We also present data on the composition of the diet of this novel, non-native population of Eastern Red-backed Salamanders in Newfoundland, which can be used to inform the scope of impact this species may have and contribute to the development of best management practices.

## Methods

### Study system

#### Study species.

The Eastern Red-backed Salamander is a small (50–120 mm, adult total length) terrestrial plethodontid salamander that is native to northeastern North America [44]. They are commonly found in and around wooded areas, often associated with damp habitats and natural cover, like leaf litter, logs, sections of bark, rocks, and an array of anthropogenic debris [44,45]. These salamanders play a vital role in maintaining the ecological balance of forested habitats, as they can achieve high biomass, drive energy flow within ecosystems through nutrient cycling, and exert strong intraguild competition [46–48]. In forests, they can reach exceptionally high biomass, representing a dominant taxon [46,49]. They can also act as a top-down regulator for the detritivore communities [48], while simultaneously influencing bottom-up effects within food webs as they are prey for numerous secondary consumers [50]. Despite their broad distribution in northeastern North America, they had not been observed before on the island of Newfoundland, however climatic niche modelling of the species’ distribution noted that the climate in Newfoundland is suitable to sustain populations [51]. Since their original description in Newfoundland, representing the first account of this species colonising a novel landscape [42], no research has examined their invasion history or ecology.

#### Study site and field collection.

Conception Bay South, Newfoundland, Canada (centred 47.5073° N, 52.9965° W) is within the Maritime Barrens Forest ecoregion, which is characterised by forests that are dominated by Balsam Firs (*Abies balsamea*), contain thick mossy forest floors, and have interspersed open heathlands [52]. This ecoregion also has cool, foggy summers and relatively mild winters [52]. The habitat and climate of this ecoregion is similar, but not identical, to the habitats within Nova Scotia, the closest native population, which are described as deciduous, coniferous, and mixed forests [53].

We collected Eastern Red-backed Salamanders (*n* = 133) from Conception Bay South during May and June of 2022. Collections involved haphazard surveys that were conducted in both anthropogenically modified habitats (e.g., backyards, gardens, lawns, and rubbish piles) and natural areas (e.g., upland forests, stream, and forested edges around wetlands). Surveys consisted of flipping cover objects (e.g., debris, logs, rocks, sections of bark, and refuse) and visually inspecting to determine if a salamander was present underneath. When an individual was found, it was captured by hand and sexed using secondary sex characteristics and candling to determine whether testes were present [54]. Age was assessed (adult or juvenile) by length, the adult threshold was a snout to posterior cloaca of at least 32 mm [55]. Salamanders were then humanely euthanized in a MS-222 (tricaine mesylate) bath, containing 500 mg/L of MS-222 (buffered with sodium bicarbonate to a pH ~ 7). Once the salamander was determined to be deceased, it was immediately preserved in 95% ethanol, then transferred to a −20°C freezer for storage until dissection.

All specimens were handled and collected under the permission of the Wildlife Division of Newfoundland and Labrador (Scientific Research Permit # WLR2022−35), and animal ethics approval through the Mount Allison University Animal Care Committee (Animal Ethics Protocol # 103181).

### Data collection

#### Prey recovery and processing.

We made an incision along the salamanders’ ventral surface to expose their stomachs whereby they were removed, longitudinally bisected, and flushed with 95% ethanol into petri dishes under a dissecting microscope. Prey items were sorted and identified to the lowest possible taxonomic level using dichotomous keys, field guides, and crowd-sourced consultation on the web-based data repository iNaturalist [56]. Each prey item was identified and categorized as either native or invasive using publicly available reference material [see: 57]. The length (L) and width (W) of every prey item was measured using a set of digital calipers to the nearest 0.01 mm and, for particularly small prey, this was done under a dissection microscope. Volume (V) of each prey item was calculated as an ellipsoid using the formula $V=\;\frac{\text{4}}{\text{3}}*\pi *L*W^{\text{2}}$ [58]. Components of prey items (e.g., legs, wings, body segments) within one sample were collated to represent a single prey item for more conservative estimates. For example, if three separate legs were recovered from the same type of prey item, and no missing legs were seen on more intact members of that taxa, then the unaccounted-for-legs were clustered and counted as a single individual. If length and width measurements were unable to be taken due to a more progressed digestive state, then data was taken from the average of that prey type from measured individuals across their level of taxonomic identification. This was done to ensure consistency and comparability.

#### Literature review.

We undertook a systematic literature review (Table S1 in S1 File) using two research repositories, ISI Web of Science Core Collection and Scopus on 11 Oct 2023 [59]. The search terms we used related to our target species’ name and dietary analysis studies were:

Web of Science: (TS=(“eastern red-backed salamander” OR “redback salamander” OR “*Plethodon cinereus*”) AND TS=(“diet*” OR “gut?content” OR “stomach?content”))

Scopus: TITLE-ABS-KEY ((“eastern red-backed salamander” OR “redback salamander” OR “*Plethodon cinereus*”) AND (“diet*” OR “gut?content” OR “stomach?content”))

We also performed a manual search of the journal Herpetological Review, as it contains natural history notes on amphibians that are not otherwise indexed within bibliographic databases. We used the ‘*find’* function (i.e., Ctrl + F) within a PDF file collation of all the available issues published between 1967 – 2023 to search for the term “*Plethodon cinereus”*. We then reviewed each article wherein the search terms were realised, and extracted dietary studies to add to the previously compiled list from the systematic review.

**Fig 1:**
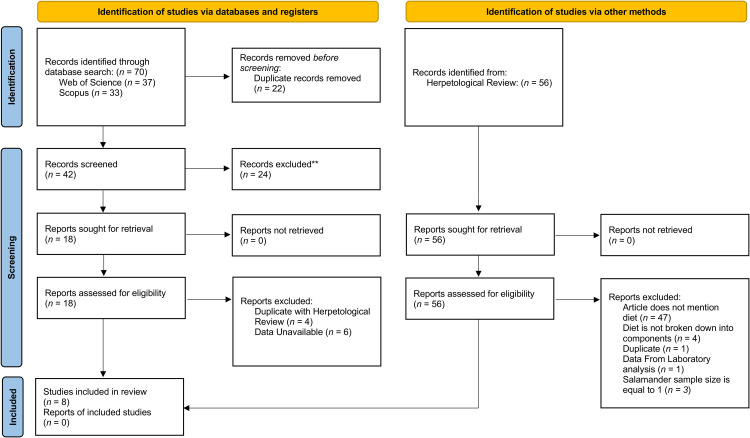
PRISMA flow diagram for data extraction in our systematic literature review with the aim to examine native range dietary niche breadth of Eastern Red-backed Salamanders.

The results of these searches were imported into Covidence systemic review software which removed duplicates [60]. Two authors (MJW and JLR) independently screened titles and abstracts. Conflicts were resolved by consensus through discussion with the third author (JBG). We reviewed 126 articles and retained studies that contained data on Eastern Red-backed Salamanders relating to: (1) general diet, (2) diet composition, (3) studies conducted on wild individuals (i.e., not in laboratory conditions), (4) those not from a duplicate article, and (5) studies that sampled more than one salamander. Using these five criteria, 117 articles were excluded and we extracted data from the 8 remaining articles (Fig 1) [61–68]. We extracted data on the count, types, and composition of prey per study location (Table S2 in S1 File). If diet data was not available in the text or supplementary materials, we contacted authors directly to obtain it. If only average prey per individual was available, we converted the value to a count of prey per study location by multiplying it by the number of individuals at the site. In one study, only graphical representations of the diet were available, so we used the package *metaDigitize* [69] in R version 4.3.2 [70] to extract data.

Across the retained articles different taxonomic levels were used for prey identification, so we standardized the taxonomic levels to allow for comparisons. This standardization, however, led to a loss of specificity. For example, where some studies included identification specificity down to the species- or genus-level, many others only identified prey down to the order- or class-level. For this reason, comparison between the Newfoundland salamander population and those from the native range are conducted primarily at the order-level; with some exceptions made for specific taxa well-known in dietary studies (e.g., separating Hymenoptera into ‘Hymenoptera’ and ‘Hymenoptera: Formicidae’ to account for bees/wasps and ants separately).

In addition to diet, we also extracted location data (i.e., latitude, longitude, and altitude) for each study site. If the locations were unavailable, they were estimated using site descriptions and approximated using Google Maps. Altitude was estimated using the US National Map – Elevation Point Query Service [71]. The purpose of examining geographic data was as a backcheck to ensure that, if the prey diversity did vary between the invasive and native population, it was not a product of a naturally occurring geographic cline in the invertebrate diversity. For example, if invertebrate diversity in the native range decreases with latitude (e.g., [72]), then diversity of the invertebrate prey in the Newfoundland salamander population may be lower regardless of native/invasive status.

### Data analysis

#### Description of diet.

Beyond a qualitative description of the stomach content of salamanders from both the native (Table S2 in S1 File) and invasive range (Table S3 in S1 File), we also calculated a Vacuity Index (i.e., the percentage of empty stomachs relative to the total number of stomachs examined multiplied by 100 [73]) for the invasive population to quantify how frequently prey was seen in salamander stomachs. We classified any prey groups that made up more than 10% in abundance or volume as a common prey group. All our analyses, including this, were completed in R version 4.3.2 [74]. To assess the factors influencing salamander diet other than whether they were invasive or not, we examined the influence of latitude, longitude, and altitude because these are potential confounding factors. As a first step, we tested the correlations between latitudinal, longitudinal, and altitude using the Spearman’s rank correlation coefficient with the function *‘rcorr’* in the *‘Hmisc’* R package [75]. There was a positive correlation between the variables latitude and longitude (*R*^*2*^ = 0.88, *p* < 0.001), a non-significant negative correlation between the variables latitude and altitude (*R*^*2*^ = −0.39, *p* = 0.10), as well as a non-significant negative correlation between the variables longitude and altitude (*R*^*2*^ = −0.35, *p* = 15). As we cannot include correlated variables within the same model, we used separate generalised linear models (GLMs) to test whether prey diversity was related to latitudinal, longitudinal, or altitude gradients using the function *‘glm’* in the ‘*glmnet’* R package [76].

#### Niche breadth invasion success hypothesis.

To test whether the diet differed between the invasive population of Eastern Red-backed Salamanders compared to conspecifics across their native range, we calculated the Shannon-Weiner diversity index for each sample using function *‘diversity’*, within the R package ‘*vegan’* [70]. We used Permutational Multivariate Analysis of Variance (PERMANOVA) with the function ‘*adonis2*’ in the ‘*vegan*’ R package to test for differences between the diets of male and female salamanders [70]. We then calculated the Pielou’s J score for each sample by dividing the Shannon-Weiner index by the *log* of the total number of prey types for the native range. We considered the estimate of diversity and evenness observed from the Newfoundland population to be significantly different from that of the native range, if the value was outside 95% confidence intervals around the native range’s average values.

We analysed the types and proportions of prey items recovered from the invasive population and recorded for native populations. We compared the original proportions in two ways: first, using a Kolmogorov-Smirnov goodness of fit test and, second, a Chi-square (χ^2^) test using the function ‘*ggbarstats*’ within the R package ‘*ggstatsplot’* [77]*.* To compare differences in proportion of the diet represented by each prey type, the counts between the native range dataset and the invasive population data were standardized by calculating the proportion and then multiplying by 100,000. This allowed for prey counts with small proportions to be preserved. Differences for each prey type were compared using χ^2^ tests using the same methods as described above [77].

#### Invasional meltdown hypothesis.

We examined the proportion of invasive and native prey within the diet of the invasive salamander population using two metrics: abundance (representing count data of individual prey items) and biomass (representing the volume of the prey consumed). Previous literature reporting invasive predator populations being supported by invasive species food items (i.e., prey or vegetation) report proportions within the gut ranging between 3–55% based on occurrence/count data [78–80] and 16–71% by volume or biomass [69,78,81]. To set our benchmark, we selected an intermediate value based on these studies (i.e., > 30% invasive prey) and examined whether the abundance of invasive vs. native prey differed from 30% invasive using a χ^2^-test. We also compared whether the biomass of the invasive vs. native prey differed from 30% invasive using a χ^2^-test.

## Results

### Description of diet

**Fig 2:**
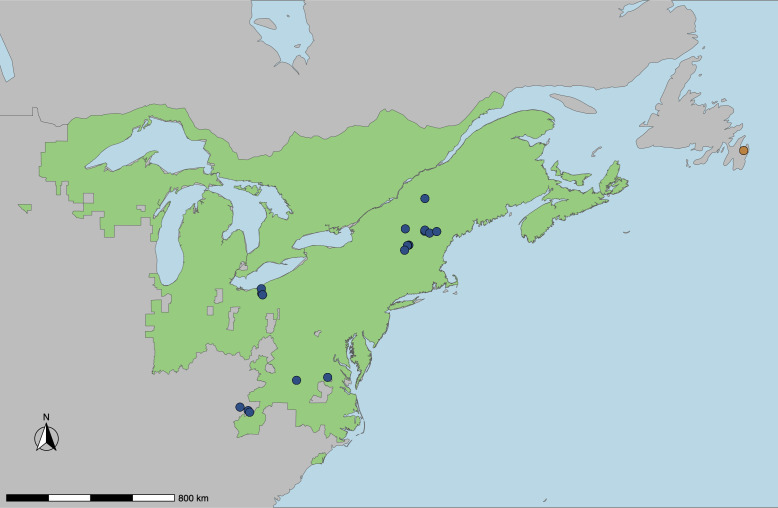
Locations of studies used in data analysis. Native range of Eastern Red-backed **Salamanders (***Plethodon cinereus***) in light green, study sites used in data collection in blue within the native range (***n* = 24), and the location of Newfoundland population in yellow (*n* = 1). Basemap was created using the R package ‘*rnaturalearth*’ [82] as well as the function ‘*geom_sf*’ from the R package ‘*ggplot2*’ [83]. *Plethodon cinereus* native range spatial data was from *the IUCN’s Red List of Threatened Species* [84].

Data on the diet of native Eastern Red-backed Salamanders was collected from 8 studies and 18 unique locations in the native range (Fig 2). In total, this dataset examined 1363 salamanders and 15,900 prey items from 25 different prey groups (plus an ‘unknown insect’ group when it could be identified to Insecta and an ‘unknown invertebrate’ for all other ambiguous items; see Table 1 for specific details on prey type and proportion of diet). Despite being considered an invertebrate generalist, two of the prey groups made up the bulk of this species’ diet in abundance, which were Acari (31.19%; mites) and Collembola (18.33%; springtails). The rest of the 25 identified prey groups made up the other half of the collated dietary dataset.

**Table 1:** Outcomes of χ^2^-test testing between diet makeup of native and invasive ranges of Eastern Red-backed Salamanders (*Plethodon cinereus*). Presented are prey groups, their common name the proportion that prey made up within the diet of the native and invasive range, as well as the *p*-values. Bold values indicate significance.

Prey Group	Common Name	Proportion within Native	Proportion within Invasive	*p* =
Arachnida: Acari	Mites	0.31191	0.18173	***<* 0.01**
Annelida	Earthworms	0.00981	0.02795	***<* 0.01**
Araneae	Spiders	0.01673	0.03541	***<* 0.01**
Archaeognatha	Bristletails	0.00006	0	***<* 0.01**
Coleoptera	Beetles	0.05069	0.10065	***<* 0.01**
Collembola	Springtails	0.18337	0.21622	***<* 0.01**
Diptera	Flies	0.03157	0.02237	***<* 0.01**
Gastropoda	Slug and Snails	0.00384	0.00839	***<* 0.01**
Hemiptera	True Bugs	0.06106	0.00932	***<* 0.01**
Hymenoptera	Sawflies, Wasps, and Bees	0.07968	0.00652	***<* 0.01**
Hymenoptera: Formicidae	Ants	0.05037	0.01864	***<* 0.01**
Insecta Unknown	Unknown Insects	0.08791	0.15564	***<* 0.01**
Isopoda	Isopods	0.00421	0.00093	***<* 0.01**
Lepidoptera	Butterflies and Moths	0.02830	0.15098	***<* 0.01**
Myriapoda	Centipedes and Millipedes	0.00019	0	***<* 0.01**
Nematoda	Nematodes	0.00164	0.01678	***<* 0.01**
Opiliones	Harvestmen	0.00006	0	***<* 0.01**
Orthoptera	Grasshoppers, Locusts, and Crickets	0.00031	0	***<* 0.01**
Plecoptera	Stoneflies	0.00755	0	***<* 0.01**
Pseudoscorpionida	Pseudoscorpions	0.00031	0	***<* 0.01**
Psocoptera	Booklice	0.00377	0	***<* 0.01**
Thysanoptera	Thrips	0.00006	0	***<* 0.01**
Trichoptera	Caddisflies	0.01327	0.01864	***<* 0.01**
Tricladida	Flatworms	0.31191	0.18173	***<* 0.01**
Unknown	Unknown	0.00981	0.02795	***<* 0.01**

In our examination of salamanders from the invasive range, we sampled a total of 133 Eastern Red-backed Salamanders (*n* = 58 females, *n* = 51 males, *n* = 24 juveniles), and or these salamanders only 3% (*n* = 4/133) were found with empty stomachs (i.e., a Vacuity Index of 3). There was no notable differences between female and male diets, supported by PERMANOVA (*F*_*1,107*_ = 0.61, *R*^*2*^ = 0.006, *p* = 0.78). Of those that contained prey items (*n* = 129), the most common prey groups in the invasive salamanders’ diet in terms of abundance were: Collembola (springtails) (21.62%), Acari (mites) (8.17%), Isopoda (isopods) (15.56%), and Chilopoda (centipedes) (10.53%). When we examine the most common prey groups by volume, they were Chilopoda (23.03%), Isopoda (19.45%), Annelida (earthworms) (16.41%), and Coleoptera (12.49%). (Table 2; see Table S3 in S1 File for the full composition of the invasive salamander diet). Overall, there was agreement that Chilopoda, Isopoda, and Annelida are major contributors to the diet of the invasive population of Eastern Red-backed Salamanders in Newfoundland.

**Table 2:** Prey items identified through dissection of the stomachs of invasive Eastern Red-backed Salamanders (*Plethodon cinereus*; *n* = 129). Prey items identified by class and order. For each prey group we present the count (n), proportion of total diet (%n), percent native prey (%N), percent invasive prey (%I), the total volume in mm^3^ (Total V), and the percent of volume compared to the whole diet (V%). Bolded volume percentages indicate a volume of that prey category that makes up over 10% of the salamander population’s total diet.

Class	Order	n	%n	%N	%I	Total V	V(%)
**Arachnida**	Araneae	38	3.54	58	42	31.12	0.83
	Ixodida	5	0.47	100	0	1.61	0.04
	Mesostigmata	139	12.95	100	0	34.09	0.91
	Opiliones	18	1.68	100	0	76.41	2.05
	Oribatida	1	0.09	100	0	0.06	0
	Sarcoptiformes	47	4.38	100	0	7.87	0.21
	Trombidiformes	2	0.19	100	0	0.64	0.02
	Unknown	2	0.19	100	0	0.4	0.01
**Arthropoda**	Unknown	8	0.75	100	0	47.27	1.27
**Chilopoda**	Geophilomorpha	24	2.24	0	100	443.08	**11.87**
	Lithobiomorpha	89	8.29	0	100	416.24	**11.15**
**Clitellata**	Enchytraeida	9	0.84	100	0	17.6	0.47
	Lumbricidae	21	1.96	0	100	594.7	**15.94**
**Diplopoda**	Julida	13	1.21	0	100	34.83	0.93
	Polydesmida	31	2.89	0	100	147.59	3.96
**Collembola**	Entomobryomorpha	149	13.89	100	0	58.68	1.57
	Poduromorph	14	1.3	100	0	3.28	0.09
	Symphypleona	66	6.15	100	0	12.54	0.34
	Unknown	2	0.19	100	0	0.43	0.01
**Gastropoda**	Stylommatophora	22	2.05	95	5	83.34	2.23
	Unknown	2	0.19	100	0	21.72	0.58
**Insecta**	Coleoptera	108	10.07	72	28	465.93	**12.49**
	Diptera	32	2.98	100	0	97.51	2.61
	Hemiptera	9	0.84	100	0	63.76	1.71
	Hymenoptera	17	1.58	100	0	29.31	0.79
	Lepidoptera	1	0.09	100	0	24.51	0.66
	Unknown	20	1.86	100	0	24.56	0.66
**Malacostraca**	Isopoda	167	15.56	2	98	725.68	**19.45**
**Symphyla**	Scolopendrellida	5	0.47	100	0	6.89	0.18
**Unknown**	Unknown	12	1.12	100	0	259.9	6.97

### Niche breadth invasion success hypothesis

We found the diversity and evenness of the salamanders’ diet to be significantly different between the native and invasive range. The native range salamanders’ diet had an average Shannon-Weiner diversity index of 2.01 (95% CI = 1.89, 2.13), whereas the invasive population’s diet had a Shannon-Weiner diversity index of 2.34. The native range salamanders’ diet had an average Pielou’s J score of 0.61 (95% CI = 0.57, 0.64) and, in contrast, the invasive populations diet had a Pielou’s J score of 0.71. Diversity scores for the native salamander populations were positively associated with latitude (*t*_16_ = 3.17, *p* = 0.006) (i.e., increasing from south to north), negatively associated with elevation (*t*_16_ = − 3.92, *p* = 0.001 (i.e., decreasing at higher elevations), and not significantly affected by longitude (*t*_16_ = 1.71, *p* = 0.107).

**Fig 3:**
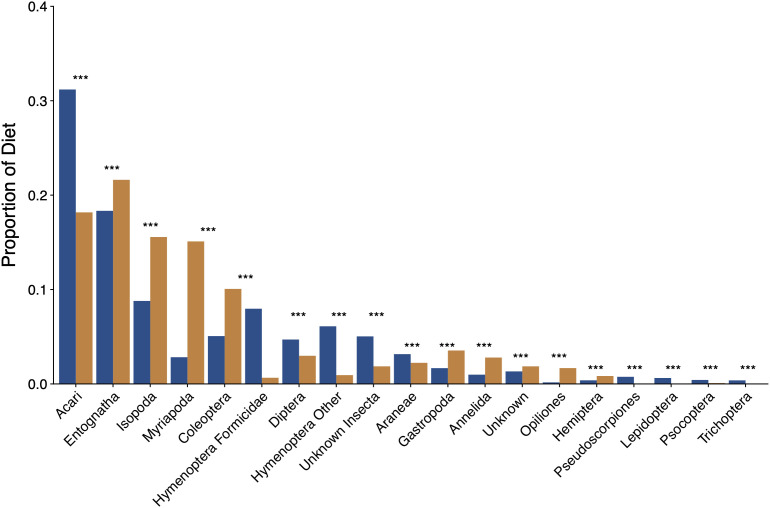
Diet proportion of top 19 prey items found in Eastern Red-backed Salamander (*Plethodon cinereus*) in native populations (*n* = 1356) through systematic literature review (brown bars) and invasive population (*n*= 129) through dissections (blue bars). Significance calculated with χ^2^-test. Significance (*p* < 0.001) is denoted by two asterisks (**).

We found that dietary composition between the native and invasive range not significantly different, based on a Kolmogorov-Smirnov goodness of fit test (*p* = 0.10). However, we found the diet significantly different based on a χ^2^-test (*x*^*2*^_*24*_ = 895.12, *p* < 0.001). There was a significant difference in the proportions of every prey item between the diets of the invasive and native populations (Table 1; Fig 3).

Overall, the invasive salamander population eats more Collembola (springtails), Isopoda (isopods), Myriapoda (centipedes and millipedes), Coleoptera (beetles), Araneae (spiders), Annelida (earthworms), Opiliones (harvestmen), and Hemiptera (true bugs) than the native population. Meanwhile, this invasive population consumed less Acari (mites), Hymenoptera: Formicidae (ants), Diptera (flies), Hymenoptera (bees and wasps; other than ants), Gastropoda (slugs and snails), Pseudoscorpionida (pseudoscopions), Lepidoptera (butterflies and moths), and Psocoptera (booklice) than native populations (Table 1). Generally comparing prey richness between the native and invasive diets, we found that 16 of the 27 identified prey groups were found within the diet of invasive salamander population, compared to the full 27 for the native (Fig 3). Those that were not seen in the invasive population were: Archaeognatha, Diplura, Homopotera, Nematoda, Orthoptera, Plecoptera, Protura, Pseudoscorpionida, Pscoptera, Thysanoptera, Trichoptera, and Turbellaria,. These prey groups, however, only represent a small proportion of the overall abundance in the native diet (see Table 1), with the combined proportion of the 14 groups absent from the invasive diet representing <2% of the overall diet in the native range.

### Invasional meltdown hypothesis

**Fig 4:**
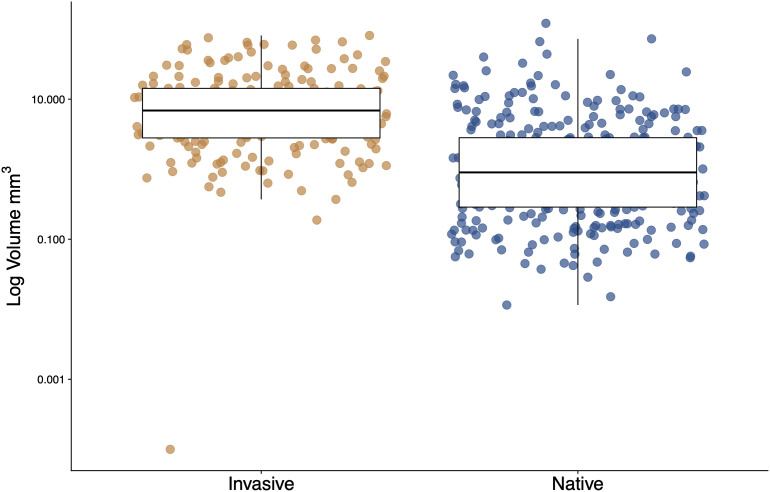
Volume, presented along a log scale, of invasive and native prey found within the diet of each invasive Eastern Red-backed Salamander (*Plethodon cinereus*). Brown points are for invasive prey items, blue points are for native prey items. Jittered raw data points are shown behind the corresponding boxplots. In the boxplots, the thick horizontal line represents the median, the boxes encompass the quartile ranges, and the whiskers represent the minimum and maximum of the data, excluding outliers (points that are 3/2 times the upper or lower quartile). A significant difference (*p* < 0.001) between native and invasive populations was calculated with χ^2^-test.

Based on abundance, the invasive salamander’s diet was made up of 63.8% native and 36.2% invasive prey, which is significantly more than our expected threshold (*x*^*2*^_*1*_ = 19.39, *p* < 0.001). The same trend is also observed when we examine the proportions of the diet based on the amount of biomass being consumed (i.e., the volume of prey). For these invasive salamanders, the total volume of their diet made up of native prey was 32.4% and invasive prey was 67.6%, which is also significantly more than our expected threshold (*x*^*2*^_*1*_ = 54.53, *p* < 0.001; Fig 4).

## Discussion

Our study provides evidence that the population of Eastern Red-backed Salamanders in Newfoundland may have undergone dietary shifts during establishment that allow this population to exploit pre-existing invasive prey communities. This foraging behaviour may have increased their ability to create niche opportunities and bolster their invasion success. We found that the invasive salamander population had a more diverse and even diet, when compared to native populations, however this could be either a product of an invasion-derived shift in diet or an extralimital continuation of the native range cline for more diverse diets at higher latitudes. Nevertheless, despite an increase in dietary diversity and evenness within the invasive population, overall dietary composition between the native and invasive range populations were not significantly different. Proportional differences in individual prey groups were detected, however, suggesting that while the general structure of the diet remains broadly similar, the relative importance of specific prey taxa differs between native and invasive range populations. This pattern partially aligns with predictions of the Niche Breadth Invasion Success Hypothesis [23]. We also found that the invasive salamander population feeds heavily on invasive prey items, both in abundance and biomass, supporting our other prediction and providing strong support for the Invasional Meltdown Hypothesis [32].

The ability to capitalize on novel resources and leverage an increasingly generalist diet is a mechanism that can allow invading populations to overcome fundamental barriers between invasion stages [14], related to acquiring enough energy to survive, reproduce, and expand [2,21,22]. In novel environments this is particularly important as the prey items that were previously relied upon within a species’ native habitat may not be readily available, and even if they, or similar prey, are present, preexisting predator-prey relationships can result in competitive exclusion by native predators. Thus, if an invading population can capitalise on a wider variety of prey items—thereby limiting the degree of competition with native taxa—then they can better integrate into a novel environment, and in doing so allocate more energy into reproduction and population growth [23]. Our findings suggest that this process may be occurring in the invasive Eastern Red-backed Salamander population in Newfoundland. The invasive population exhibited a more diverse and more even diet compared with native populations. Yet, our analysis also indicates that dietary diversity within the native range is associated with geographic clines in latitude and elevation, with dietary diversity increasing across their native range in more northern and lowland populations. Given that our study represents the most northerly examined population (Fig 2), this finding could be simply an extension of the native range cline. However, we posit this still may represent a prior adaptation that could provide an invasion advantage if the origin population is also from a northern latitude (e.g., the Canadian Maritimes) which preliminary molecular analyses suggest [85]. To disentangle whether the increased dietary diversity is a product of invasion-derived change or a prior adaption resulting from a natural cline will require further comparative studies between the specific origin population and the invasive range. Nevertheless, we saw that the invasive salamander’s diet shifted away from feeding heavily on Acari (mites) and, instead, feeding on a wider variety of prey, notably more Isopoda (wood lice), Myriapoda (centipedes and millipedes), and Coleoptera (beetles) (see Supplementary Materials for details on changes in prey composition). The specifics of our observed dietary shift (i.e., a diet higher in proportions of Isopoda, Myriapoda, and Coleoptera) has previously been seen in other invasive amphibian populations [86,87]. The invasive population of Guttural Toads (*Sclerophrys gutturalis*) in Mauritius significantly increased the portion of isopods consumed compared to native populations [87,88] and invasive Coquí Frog (*Eleutherodactylus coqui*) population in Hawai’i were found to have prey preferences towards isopods, coleopterans, and myriapods [86]. This may suggest that these invertebrate taxa incur less predation pressure from native specialist predators in these habitats, allowing for these invasive dietary shifts to more readily occur, however more research into these dynamics are needed.

We found that the invasive population contained 16 of the 25 prey groups found within the diet of the native salamander populations (see Supplementary Materials for details). Yet, most of these unseen prey groups in the invasive diet, only constituted fractional proportions of the overall diet in the native range, and given the wide geographic range of the native range population a higher species richness would be expected. The changes in the proportion of major prey groups seen in the invasive population, compared to that of the native populations, is likely the driving force increasing the diversity of prey eaten by Eastern Red-backed Salamanders in Newfoundland. This supposition is also supported by the fact that this increasingly generalist diet resulted in more evenness between major prey groups. Together, this supports our predictions that the diet of the invasive salamander would increase in species richness and dietary niche breadth, resulting in a shift towards a more generalist diet. Such modulations can also be seen in populations that are adapting to urban environments—another form of novel landscape/ecosystem colonisation—whereby the ability to broaden a population’s dietary niche breadth may increase urban survival [89,90]. Dietary niche broadening is not always the response of invasive species. For example, the American Bullfrog, a generalist species native to North America, but invasive across a wide global range, exhibits a significantly narrower dietary niche in its invasive populations compared to those from its native range [78], focusing on the most abundant prey and reducing its trophic strategy and niche breadth. It is likely that an invasive population’s dietary response, whether broadening or constricting its niche, depends on prey availability, competition, and the drive for resource optimization. Regardless of the directionality (i.e., broadening or narrowing), however, the ability to shift dietary niche breadth adaptively has the potential to provide colonising populations the means to gain necessary resources they may otherwise be excluded from. Another mechanism for increasing access to dietary resources, by reducing potential competition, could result from invaders actively feeding on prey that do not share a recent evolutionary history with native predators (i.e., invasive prey items).

High functioning ecosystems, such as those with much of their available niches filled, are often more difficult to invade [29], while disrupted or disturbed ecosystems are often seen to be less resistant to invasion [30,91]. Our findings regarding the high proportion of invasive prey in the salamander’s diet, in both abundance (36.2% by count) and biomass (67.6% by volume) suggests that previously established invasive invertebrate communities may have bolstered the successful establishment and spread of Eastern Red-backed Salamanders in Newfoundland. This disparity in dietary composition based on count and volume is driven by the size differences between prey types, with native invertebrates, like Acari (mites) and Collembola (springtails), being consumed in high numbers but contributing little to overall volume, while larger invasive prey, like Annelida (earthworms), contribute significantly more despite being less frequent. Thus, the majority of energy fuelling this invasive population of salamanders is derived from prey that similarly have not evolved within the ecosystem and thus may be underexploited in the region due to the potential absence of their own evolved predators [19]. This finding suggests that the previous invasions of invertebrates to Newfoundland may have created an open niche, or at least increased niche opportunities, for invading Eastern Red-backed Salamanders to capitalise upon. Similarly, invasive American Bullfrog populations in China preferentially prey on invasive crayfish [79] and a large proportion of the diet of invasive Coquí Frogs in Hawai’i consists of invasive termites and ants [86]. In general, these studies, as well as our own presented here, suggests the presence of pre-existing invasive prey can create novel niche opportunities for incoming invaders to capitalise on. This also means that anthropogenic activities that promote the spread and establishment of some invasive species, including some that many may consider benign or inconsequential (e.g., isopods and millipedes), can have knock-on effects that promote the invasive potential for other taxa that might be seen as more ecologically or economically hazardous (e.g., invasive amphibians).

Although our findings suggest that the invasive Eastern Red-backed Salamanders in Newfoundland have altered their diets, compared to native conspecific populations, there remains a clear need for significantly more research on this system. A particular limitation of this study is that little is known about the invasive invertebrate communities in Newfoundland (e.g., density, abundance, invasion history, and ecological role and impact). Such information would provide important clarity regarding feeding choice and selection. Information on prey availability and diet composition could then be used to calculate a Relativized Electivity Index to examine the salamanders’ dietary choices in relation to the abundance and nutritional value of available prey items [87,88]. By analysing the prey availability on the island, this would shed light on whether salamanders are actively selecting larger, more nutrient-dense invasive prey or simply consuming them due to their higher abundance and frequent encounter rates. Additionally, soft-bodied prey items are less likely to be uncovered in diet analysis studies due to their level of digestion and loss of identifiable characteristics. By performing a molecular analysis of the dietary samples, a higher level of specificity could be achieved for highly digested or incomplete prey items [92]. We also suggest investigating nutritional shifts in prey items, such as comparing the caloric content of Acari (mites) versus Isopoda (isopods), Myriapoda (centipedes and millipedes), and Coleoptera (beetles). Finally, more taxonomically detailed examinations of diet within the salamander’s native range, including comparison of native and invasive prey items would allow for more accurate comparisons with the invasive salamander population. Such studies could elucidate the drivers behind dietary changes observed in invasive salamander populations. These important next steps will provide valuable context to aid in our understanding of how dietary niche flexibility can enhance a species’ invasive potential and allow them to overcome barriers when colonising new habitats.

### Conclusion

At the core of all biological invasions is human activity; both in the spread of invasive species [13] and the disturbance of natural areas and ecological processes. These activities create ecological opportunities for invaders to establish [30,31,91]. Human activities, such as urbanization and ever-growing transport networks, can lead to an increase in the invasive invertebrate populations forming [93], which, as we posit here, may bolster invasion success of generalist invertebrate predators. This challenges the concept of a harmless or inconsequential invasive species (i.e., those that do not appear to be directly negatively impacting native ecosystems). The success of these seemingly harmless taxa may indirectly pose a threat by creating exploitable dietary niche opportunities for future invasive predators. This is pertinent, as invasive invertebrates make up one of the largest proportions of invasive taxa globally, second only to plants [94,95]. Due to the increasing numbers of invasive invertebrates around the world, and the ability for generalist vertebrate predators, like Eastern Red-backed Salamander, to feed on them, we may expect to see even more invasive predator species continue to spread; using invasive invertebrate prey communities as a springboard to strengthen their invasive potential.

## Supporting information

S1 File Supplementary Materials_Williams et al. This one file contains Table S1 that is the PRISMA checklist for the manuscript, Table S2 which details the data extracted during our systematic literature review about the diet of Eastern Red-backed Salamanders in their native range, and, lastly, a detailed list of the different taxa recovered from Eastern Red-backed Salamanders in their invasive range in Table S3.
(PDF)
