# Supplementary material for: In the belly of the beast: How dietary changes and preexisting invasive prey may have promoted the success of a novel invasive amphibian
Source: PLoS One. 2026 Jul 10;21(7):e0352970. doi: 10.1371/journal.pone.0352970 (PMC13354068; doi:10.1371/journal.pone.0352970)
Supplement: S1 File — This one file contains Table S1 that is the PRISMA checklist for the manuscript, Table S2 which details the data extracted during our systematic literature review about the diet of Eastern Red-backed Salamanders in their native range, and, lastly, a detailed list of the different taxa recovered from Eastern Red-backed Salamanders in their invasive range in Table S3. (PDF) [file pone.0352970.s001.pdf]

Supplementary materials for ***"In the Belly of the Beast: How Dietary Changes and Preexisting Invasive Prey may have Promoted the Success of a Novel Invasive Amphibian"*** by Maya J. Williams, Julia L. Riley, and James Baxter-Gilbert

As additional materials, we have included a PRISMA checklist to outline the process that we undertook for our systematic literature review (S1 Table), the data extracted during our systematic literature review about the diet of Eastern Red-backed Salamanders in their native range (S2 Table), and a detailed list of all of the different taxa (identified to the lowest taxonomic level possible) that was recovered from the stomach contents of the invasive Eastern Red-backed Salamanders we collected from Newfoundland (S3 Table).

S1 Table. PRISMA checklist for manuscript.

Section and Topic	Item #	Checklist item	Location where item is reported
**TITLE**			
Title	1	Identify the report as a systematic review.	In the Keywords pg. 1
**ABSTRACT**			
Abstract	2	See the PRISMA 2020 for Abstracts checklist.	pg. 2
**INTRODUCTION**			
Rationale	3	Describe the rationale for the review in the context of existing knowledge.	pg. 8
Objectives	4	Provide an explicit statement of the objective(s) or question(s) the review addresses.	pg. 9
**METHODS**			
Eligibility criteria	5	Specify the inclusion and exclusion criteria for the review and how studies were grouped for the syntheses.	pg. 12
Information sources	6	Specify all databases, registers, websites, organisations, reference lists and other sources searched or consulted to identify studies. Specify the date when each source was last searched or consulted.	pg. 12
Search strategy	7	Present the full search strategies for all databases, registers and websites, including any filters and limits used.	pg. 12
Selection process	8	Specify the methods used to decide whether a study met the inclusion criteria of the review, including how many reviewers screened each record and each report retrieved, whether they worked independently, and if applicable, details of automation tools used in the process.	pg. 13
Data collection process	9	Specify the methods used to collect data from reports, including how many reviewers collected data from each report, whether they worked independently, any processes for obtaining or confirming data from study investigators, and if applicable, details of automation tools used in the process.	pg. 13
Data items	10a	List and define all outcomes for which data were sought. Specify whether all results that were compatible with each outcome domain in each study were sought (e.g. for all measures, time points, analyses), and if not, the methods used to decide which results to collect.	pg. 13-14
	10b	List and define all other variables for which data were sought (e.g. participant and intervention characteristics, funding sources). Describe any assumptions made about any missing or unclear information.	pg. 13-14
Study risk of bias assessment	11	Specify the methods used to assess risk of bias in the included studies, including details of the tool(s) used, how many reviewers assessed each study and whether they worked independently, and if applicable, details of automation tools used in the process.	pg. 12
Effect measures	12	Specify for each outcome the effect measure(s) (e.g. risk ratio, mean difference) used in the synthesis or presentation of results.	NA

Section and Topic	Item #	Checklist item	Location where item is reported
Synthesis methods	13a	Describe the processes used to decide which studies were eligible for each synthesis (e.g. tabulating the study intervention characteristics and comparing against the planned groups for each synthesis (item #5)).	pg. 13
	13b	Describe any methods required to prepare the data for presentation or synthesis, such as handling of missing summary statistics, or data conversions.	pg. 13
	13c	Describe any methods used to tabulate or visually display results of individual studies and syntheses.	pg. 13
	13d	Describe any methods used to synthesize results and provide a rationale for the choice(s). If meta-analysis was performed, describe the model(s), method(s) to identify the presence and extent of statistical heterogeneity, and software package(s) used.	pg. 13
	13e	Describe any methods used to explore possible causes of heterogeneity among study results (e.g. subgroup analysis, meta-regression).	NA
	13f	Describe any sensitivity analyses conducted to assess robustness of the synthesized results.	NA
Reporting bias assessment	14	Describe any methods used to assess risk of bias due to missing results in a synthesis (arising from reporting biases).	NA
Certainty assessment	15	Describe any methods used to assess certainty (or confidence) in the body of evidence for an outcome.	pg. 15
**RESULTS**			
Study selection	16a	Describe the results of the search and selection process, from the number of records identified in the search to the number of studies included in the review, ideally using a flow diagram.	pg. 13
	16b	Cite studies that might appear to meet the inclusion criteria, but which were excluded, and explain why they were excluded.	pg. 13
Study characteristics	17	Cite each included study and present its characteristics.	pg. 13
Risk of bias in studies	18	Present assessments of risk of bias for each included study.	NA
Results of individual studies	19	For all outcomes, present, for each study: (a) summary statistics for each group (where appropriate) and (b) an effect estimate and its precision (e.g. confidence/credible interval), ideally using structured tables or plots.	Table SM2
Results of syntheses	20a	For each synthesis, briefly summarise the characteristics and risk of bias among contributing studies.	Table SM2
	20b	Present results of all statistical syntheses conducted. If meta-analysis was done, present for each the summary estimate and its precision (e.g. confidence/credible interval) and measures of statistical heterogeneity. If comparing groups, describe the direction of the effect.	pg. 15
	20c	Present results of all investigations of possible causes of heterogeneity among study results.	NA
	20d	Present results of all sensitivity analyses conducted to assess the robustness of the synthesized results.	NA
Reporting biases	21	Present assessments of risk of bias due to missing results (arising from reporting biases) for each synthesis assessed.	NA
Certainty of evidence	22	Present assessments of certainty (or confidence) in the body of evidence for each outcome assessed.	pg. 15
**DISCUSSION**			
Discussion	23a	Provide a general interpretation of the results in the context of other evidence.	pg. 22

Section and Topic	Item #	Checklist item	Location where item is reported
	23b	Discuss any limitations of the evidence included in the review.	pg. 22
	23c	Discuss any limitations of the review processes used.	NA
	23d	Discuss implications of the results for practice, policy, and future research.	pg. 26 - 27
**OTHER INFORMATION**			
Registration and protocol	24a	Provide registration information for the review, including register name and registration number, or state that the review was not registered.	pg. 27
	24b	Indicate where the review protocol can be accessed, or state that a protocol was not prepared.	See methods
	24c	Describe and explain any amendments to information provided at registration or in the protocol.	NA
Support	25	Describe sources of financial or non-financial support for the review, and the role of the funders or sponsors in the review.	pg. 28
Competing interests	26	Declare any competing interests of review authors.	pg. 27
Availability of data, code and other materials	27	Report which of the following are publicly available and where they can be found: template data collection forms; data extracted from included studies; data used for all analyses; analytic code; any other materials used in the review.	pg. 27

S2 Table. Sources from which data was extracted to examine native range dietary niche breadth of Eastern Red-back Salamanders. The paper's citation (as per the bibliography in the main manuscript), diet extraction method, geographic area, latitudes, longitudes, sample size (n), Shannon-Wiener Index, and Pielous'J are presented for each article.

Citation	Extraction Method	Geographic Area	Latitude	Longitude	*n*	Shannon-Wiener Index	Pielou's J
[1]	Gastric Lavage	Ohio, USA	41.286667	-81.571833	81	1.96	0.71
[2]	Dissection	Virginia, USA	37.523611	-79.506111	15	1.74	0.72
[3]	Gastric Lavage	New Hampshire, USA	43.90723	-71.61177	31	1.88	0.66
			43.97367	-71.18802	32	2.05	0.72
			43.98785	-71.90552	24	1.90	0.67
			44.03436	-71.89082	25	1.93	0.68
		Vermont, USA	43.16293	-73.09455	32	1.79	0.63
			43.36156	-72.93236	24	1.68	0.59
			43.36733	-72.9128	23	1.74	0.61
			43.38507	-72.84328	13	1.71	0.60
			44.09015	-73.05123	11	1.66	0.58
[4]	Gastric Lavage	Ohio, USA	41.492694	-81.592667	85	1.83	0.63
[5]	Gastric Lavage	Tennessee, USA	36.137667	-82.287917	135	1.49	0.62
			36.211167	-82.3765	109	1.76	0.74
			36.361583	-82.860472	104	1.74	0.73
[6]	Gastric Lavage	Virginia, USA	37.651972	-77.658333	30	1.77	0.67
[7]	Gastric Lavage	Quebec, Canada	45.404444	-71.888333	30	2.12	0.69
[8]	Gastric Lavage	Ohio, USA	41.229617	-81.518825	256	2.07	0.73

S3 Table. Prey items identified through dissection of the stomachs of *Plethodon cinereus* (*n* = 129/133). Prey items were identified by class, order, family, genus, and species where possible. For each prey group, we present the number of prey items (*n*), then if the prey item is considered native yes or no, and the percent volume the prey item made up of the whole diet of all salamanders sampled (V%).

Class	Order	Family	*Genus*	*Species*	*n*	Native (Y or N)	%V
**Arachnida**	Araneae	Clubionidae			1	Y	0.007
**Arachnida**	Araneae	Linyphiidae	*Pityohyphantes*		2	Y	0.021
**Arachnida**	Araneae	Linyphiidae	*Tenuiphantes*		1	Y	0.009
**Arachnida**	Araneae	Linyphiidae			6	Y	0.103
**Arachnida**	Araneae	Miturgidae	*Chiracanthium*	*iclusum*	1	Y	0.004
**Arachnida**	Araneae	Pholcidae			10	N	0.227
**Arachnida**	Araneae	Theridiidae	*Enoplognatha*	*ovata*	6	N	0.178
**Arachnida**	Araneae	Unknown			11	Y	0.286
**Arachnida**	Ixodida	Ixodidae	*Haemaphysalis*	*leporispalustris*	2	Y	0.013
**Arachnida**	Ixodida	Ixodidae			1	Y	0.009
**Arachnida**	Ixodida				2	Y	0.021
**Arachnida**	Mesostigmata				138	Y	0.909
**Arachnida**	Opiliones	Nemastomatidae	*Nemastoma*	*lugubre*	3	Y	0.186
**Arachnida**	Opiliones	Phalangiidae	*Oligolophus*	*hansenii*	2	Y	0.191
**Arachnida**	Opiliones	Phalangiidae	*Phalangium*	*opilio*	1	Y	0.040
**Arachnida**	Opiliones	Phalangiidae	*Rilaena*	*triangularis*	2	Y	0.469
**Arachnida**	Opiliones	Phalangiidae	*Rilaena*	*triangularis*	1	Y	0.366
**Arachnida**	Opiliones	Phalangiidae			9	Y	0.796
**Arachnida**	Oribatida				1	Y	0.002
**Arachnida**	Sarcoptiformes				47	Y	0.211
**Arachnida**	Trombidiformes				2	Y	0.017
**Arachnida**	Unknown				2	Y	0.011
**Arthropod**	Unknown				8	Y	1.267
**Chilopoda**	Geophilomorpha	Himantariidae	*Haplophilus*	*subterraneus*	3	N	0.584
**Chilopoda**	Geophilomorpha	Himantariidae	*Haplophilus*	*subterraneus*	19	N	9.942
**Chilopoda**	Geophilomorpha	Himantariidae			2	N	1.348
**Chilopoda**	Lithobiomorpha	Lithobiidae	*Lithobius*	*forficatus*	84	N	10.937
**Chilopoda**	Lithobiomorpha	Lithobiidae	*Lithobius*	*microps*	2	N	0.069
**Chilopoda**	Lithobiomorpha	Lithobiidae			2	N	0.060
**Chilopoda**	Lithobiomorpha				1	N	0.088
**Clitellata**	Enchytraeida	Enchytraeidae			9	Y	0.472
**Clitellata**	Lumbricidae	Lumbricidae	*Lumbricus*	*terrestris*	21	N	15.937
**Diplopoda**	Julida	Blaniulidae			8	N	0.427
**Diplopoda**	Julida	Julidae	*Brachyiulus*		3	N	0.362
**Diplopoda**	Julida	Julidae	*Cylindroiulus*	*punctatus*	2	N	0.145

**Diplopoda**	Polydesmida	Polydesmidae	*Polydesmus*	*angustus*	16	N	2.496
**Diplopoda**	Polydesmida	Polydesmidae	*Polydesmus*		1	N	0.060
**Diplopoda**	Polydesmida	Polydesmidae			14	N	1.399
**Entognatha**	Entomobryomorpha	Entomobryidae	*Entomobrya*	*nivalis*	1	Y	0.069
**Entognatha**	Entomobryomorpha				148	Y	1.504
**Entognatha**	Mesostigmata				1	Y	0.004
**Entognatha**	Poduromorph	Hypogastruridae			1	Y	0.004
**Entognatha**	Poduromorph	Neanuridae			8	Y	0.052
**Entognatha**	Poduromorph				5	Y	0.032
**Entognatha**	Symphypleona	Arthopalitidae			1	Y	0.001
**Entognatha**	Symphypleona	Dicyrtomidae			51	Y	0.267
**Entognatha**	Symphypleona	Dicyrtomidae	*Dicyrtoma*	*flammea*	2	Y	0.020
**Entognatha**	Symphypleona	Sminthurididae			1	Y	0.018
**Entognatha**	Symphypleona				11	Y	0.030
**Entognatha**	Unknown				2	Y	0.011
**Gastropoda**	Stylommatophora	Agriolimacidae			1	Y	0.455
**Gastropoda**	Stylommatophora	Gastrodontidae	*Perpolita*		4	Y	0.322
**Gastropoda**	Stylommatophora	Hygromiidae	*Trochulus*	*hispidus*	1	N	0.057
**Gastropoda**	Stylommatophora	Vitrinidae	*Vitrina*		3	Y	0.241
**Gastropoda**	Stylommatophora	Vitrinidae			1	Y	0.022
**Gastropoda**	Stylommatophora				12	Y	1.136
**Gastropoda**	Unknown				2	Y	0.582
**Insecta**	Coleoptera	Carabidae	*Clivina*	*fossor*	1	N	0.103
**Insecta**	Coleoptera	Carabidae	*Harpalus*	*rufipes*	2	N	0.516
**Insecta**	Coleoptera	Carabidae	*Pterostichus*		2	Y	1.056
**Insecta**	Coleoptera	Chrysomelidae	*Chrysolina*	*hyperici*	1	N	0.032
**Insecta**	Coleoptera	Chrysomelidae			3	N	0.090
**Insecta**	Coleoptera	Cryptophagidae	*Cryptophagus*		2	Y	0.133
**Insecta**	Coleoptera	Curculionidae	*Barypeithes*	*pellucidus*	3	N	0.355
**Insecta**	Coleoptera	Curculionidae	*Dendroctonus*		1	Y	0.076
**Insecta**	Coleoptera	Curculionidae	*Glocianus*		1	N	0.117
**Insecta**	Coleoptera	Curculionidae	*Hypera*	*nigrirostris*	2	N	0.424
**Insecta**	Coleoptera	Curculionidae	*Isochnus*	*sequensi*	1	N	0.035
**Insecta**	Coleoptera	Curculionidae	*Orthochaetes*	*setiger*	1	N	0.038
**Insecta**	Coleoptera	Curculionidae	*Otiorhynchus*	*porcatus*	1	N	0.596
**Insecta**	Coleoptera	Curculionidae	*Phyllobius*	*oblongus*	5	N	0.737
**Insecta**	Coleoptera	Curculionidae	*Phyllobius*	*oblongus*	2	N	0.149
**Insecta**	Coleoptera	Curculionidae	*Sitona*	*hispidulus*	2	N	0.174
**Insecta**	Coleoptera	Curculionidae	*Tychius*		2	N	0.090

**Insecta**	Coleoptera	Curculionidae			5	Y	0.580
**Insecta**	Coleoptera	Elateridae	*Agriotes*	*ineatus*	1	N	0.169
**Insecta**	Coleoptera	Elateridae	*Agriotes*	*mancus*	1	N	0.804
**Insecta**	Coleoptera	Hydrophilidae	*Anacaena*	*limbata*	1	N	0.077
**Insecta**	Coleoptera	Latridiidae			3	Y	0.014
**Insecta**	Coleoptera	Staphylinidae	*Palporus*	*nitidulus*	5	Y	0.099
**Insecta**	Coleoptera	Staphylinidae	*Stenus*		2	Y	0.074
**Insecta**	Coleoptera	Staphylinidae			44	Y	5.586
**Insecta**	Coleoptera				14	Y	0.361
**Insecta**	Diptera	Anisopodidae	*Sylvicola*	*fenestralis*	2	Y	0.049
**Insecta**	Diptera	Calliphoridae	*Calliphora*		1	Y	0.083
**Insecta**	Diptera	Calliphoridae			1	Y	0.077
**Insecta**	Diptera	Culicidae			1	Y	0.003
**Insecta**	Diptera	Cyclorrhapha			1	Y	1.947
**Insecta**	Diptera	Phoridae			4	Y	0.111
**Insecta**	Diptera	Sciaridae			21	Y	0.307
**Insecta**	Diptera	Sphaeroceridae			1	Y	0.037
**Insecta**	Hemiptera	Rhyparochromidae	*Drymus*	*unus*	6	Y	1.073
**Insecta**	Hemiptera	Rhyparochromidae			2	Y	0.458
**Insecta**	Hemiptera				1	Y	0.177
**Insecta**	Hymenoptera	Chrysidoidea			1	Y	0.007
**Insecta**	Hymenoptera	Fanniidae			1	Y	0.011
**Insecta**	Hymenoptera	Formicidae	*Formica*	*glacialis*	1	Y	0.161
**Insecta**	Hymenoptera	Formicidae	*Myrmica*	*detritinodis*	6	Y	0.431
**Insecta**	Hymenoptera	Pergidae	*Acordulecera*	*dorsalis*	1	Y	0.151
**Insecta**	Hymenoptera	Tenthredinidae			1	Y	0.003
**Insecta**	Hymenoptera				6	Y	0.021
**Insecta**	Lepidoptera	Noctuidae	*Nephelodes*	*minians*	1	Y	0.657
**Insecta**	Unknown				20	Y	0.658
**Malacostraca**	Isopoda	Porcellionidae	*Porcellio*	*scaber*	161	N	18.679
**Malacostraca**	Isopoda	Porcellionidae			2	N	0.110
**Malacostraca**	Isopoda	Trichoniscidae	*Trichoniscus*	*pusillus*	2	Y	0.421
**Malacostraca**	Isopoda				2	Y	0.237
**Symphyla**	Scolopendrellida	Scutigerellidae	*Scutigerella*		5	Y	0.185
**Unknown**	Unknown				12	Y	6.965

Bibliography

[1] Anthony CD, Venesky MD, Hickerson CA. Ecological separation in a polymorphic terrestrial salamander. J Anim Ecol. 2008;77(4):646–53. doi: 10.1111/j.1365-2656.2008.01398.x

[2] Arif S, Adams D, Wicknick J. Bioclimatic modelling, morphology, and behaviour reveal alternative mechanisms regulating the distributions of two parapatric salamander species. Evol Ecol Res. 2007;9(5):843-854.

[3] Bondi CA, Beier CM, Fierke MK, Ducey PK. The role of feeding strategy in the tolerance of a terrestrial salamander (*Plethodon cinereus*) to biogeochemical changes in northern hardwood forests. Can J Zool. 2019;97(4):281-93. doi: 10.1139/cjz-2017-0302

[4] Ivanov K, Lockhart OM, Keiper J, Walton BM. Status of the exotic ant *Nylanderia flavipes* (Hymenoptera: Formicidae) in northeastern Ohio. Biol Invasions. 2011;13:1945-50. doi: 10.1007/s10530-011-0021-z

[5] Maglia AM. Ontogeny and feeding ecology of the Red-backed Salamander, *Plethodon cinereus*. Copeia. 1996;1996:576-586. doi: 10.2307/1447521

[6] Mitchell JC, Woolcott WS. Observations of the microdistribution, diet and predator-prey size relationships in the salamander *Plethodon cinereus* from the Virginia Piedmont (USA). Virginia J Sci. 1985;36:281-88.

[7] Scott T, Bradley RL, Bourgault P. Non-native earthworms increase the abundance and diet quality of a common woodland salamander in its northern range. Biol Invasions. 2024;26(1):187-200. doi: 10.1007/s10530-023-03168-3

[8] Stuczka A, Hickerson CA, Anthony C. Niche partitioning along the diet axis in a colour polymorphic population of Eastern Red-backed Salamanders, *Plethodon cinereus*. Amphibia-Reptilia. 2016;37(3):283-90.
