# Supplementary material for: In the belly of the beast: How dietary changes and preexisting invasive prey may have promoted the success of a novel invasive amphibian
Source: PLoS One. 2026 Jul 10;21(7):e0352970. doi: 10.1371/journal.pone.0352970 (PMC13354068; doi:10.1371/journal.pone.0352970)
Supplement: Attachment — Submitted filename: Response to Reviewers_Williams et al_final.docx [file pone.0352970.s002.docx]

**Responses to Reviewers**

*Comments are in bold with the responses in plain text immediately below.*

**Academic Editor**

**Journal Requirements**

**When submitting your revision, we need you to address these additional requirements:**

**1. Please ensure that your manuscript meets PLOS ONE's style requirements, including those for file naming. The PLOS ONE style templates can be found at**

[**https://journals.plos.org/plosone/s/file?id=wjVg/PLOSOne_formatting_sample_main_body.pdf**](https://journals.plos.org/plosone/s/file?id=wjVg/PLOSOne_formatting_sample_main_body.pdf) **and**

[**https://journals.plos.org/plosone/s/file?id=ba62/PLOSOne_formatting_sample_title_authors_affiliations.pdf**](https://track.editorialmanager.com/CL0/https:%2F%2Fjournals.plos.org%2Fplosone%2Fs%2Ffile%3Fid=ba62%2FPLOSOne_formatting_sample_title_authors_affiliations.pdf/1/010f01985636c793-9deede4f-c144-4b88-b048-f5184746ecc4-000000/9gVZbQYtDIx0olEllNsrC3F2VwL9MTbSnflTJ7B6sDg=221)

We have now gone through and revised the formatting following the link you have provide here. Thank you for sharing them and we hope our manuscript in now aligned with the PLOS ONE formatting style.

**2. Thank you for stating the following financial disclosure:
1)	Author's Initials: M. J. W.**

**Award Name: J. E. A. Crake Foundation Grant**

**Funder Names: Mount Allison University**

**2)	Author's Initials: J. B-G.**

**Award Name: Part-time Research Stipend**

**Funder Names: Mount Allison University**

**3)	Author's Initials: J. L. R.**

**Award Name: Discovery Grant**

**Funder Names: Natural Sciences and Engineering Research Council of Canada (NSERC)**

**Please state what role the funders took in the study. If the funders had no role, please state: "****The funders had no role in study design, data collection and analysis, decision to publish, or preparation of the manuscript." If this statement is not correct you must amend it as needed. Please include this amended Role of Funder statement in your cover letter; we will change the online submission form on your behalf.**

We have now included the statement “The funders had no role in study design, data collection and analysis, decision to publish, or preparation of the manuscript." in the cover letter and have also included it in the Funding section of the manuscript.

**3. We note that Figure 2 in your submission contain map images which may be copyrighted. All PLOS content is published under the Creative Commons Attribution License (CC BY 4.0), which means that the manuscript, images, and Supporting Information files will be freely available online, and any third party is permitted to access, download, copy, distribute, and use these materials in any way, even commercially, with proper attribution. For these reasons, we cannot publish previously copyrighted maps or satellite images created using proprietary data, such as Google software (Google Maps, Street View, and Earth). For more information, see our copyright guidelines:** [**http://journals.plos.org/plosone/s/licenses-and-copyright**](http://journals.plos.org/plosone/s/licenses-and-copyright)**.**

Thank you for your keen attention to detail in ensuring copyrighted work. We would like to clarify that the map we created for Figure 2 was made using open-source data. We used the R package ‘*rnaturalearth*’ to collect the data for our map. This package utilizes publicly available data – from Natural Earth, a source that is fully open and accessible to everyone. We then used the *‘geom_sf’* function from the R package ‘*ggplot2*’ to generate the basemap. To give proper credit, we have referenced both packages used in the figure caption. In short, this figure is not previously copyrighted maps or satellite images, nor is it made from data that is.

**4. Please include captions for your Supporting Information files at the end of your manuscript, and update any in-text citations to match accordingly. Please see our Supporting Information guidelines for more information:** [**http://journals.plos.org/plosone/s/supporting-information**](http://journals.plos.org/plosone/s/supporting-information)**.**

We have now updated the Supporting Information file and in-text citations to match the suggested format for having one file with multiple tables within it.

**5. Please remove all personal information, ensure that the data shared are in accordance with participant consent, and re-upload a fully anonymized data set.**

**Note: spreadsheet columns with personal information must be removed and not hidden as all hidden columns will appear in the published file.**

**Additional guidance on preparing raw data for publication can be found in our Data Policy (**[**https://journals.plos.org/plosone/s/data-availability#loc-human-research-participant-data-and-other-sensitive-data**](https://journals.plos.org/plosone/s/data-availability#loc-human-research-participant-data-and-other-sensitive-data)**) and in the following article:** [**http://www.bmj.com/content/340/bmj.c181.long**](http://www.bmj.com/content/340/bmj.c181.long)**.**

Thank you for your comment. All third-party data collected for our literature review has been cited both in our paper, the supplementary materials, and in the data file. We understand the importance of not publishing personal information and have removed any personal information from our data set. We also made sure that there are no hidden columns in our data set. If we have missed any specific information you wish to be removed, please let us know.

**6. If the reviewer comments include a recommendation to cite specific previously published works, please review and evaluate these publications to determine whether they are relevant and should be cited. There is no requirement to cite these works unless the editor has indicated otherwise.**

Thank you. We understand and have read and cited appropriate work.

**7. Please review your reference list to ensure that it is complete and correct. If you have cited papers that have been retracted, please include the rationale for doing so in the manuscript text, or remove these references and replace them with relevant current references. Any changes to the reference list should be mentioned in the rebuttal letter that accompanies your revised manuscript. If you need to cite a retracted article, indicate the article’s retracted status in the References list and also include a citation and full reference for the retraction notice.**

Thank you. We have gone through all our cited literature and our literature review list. There were no papers that were retracted. If you are aware of a specific instance and we have missed it, please let us know.

**General Comment**

**1. I have one minor issue that I think could be addressed. I believe the authors should include a paragraph stating the limitations of the study. I say this in the sense that:**

**First: Diet is often linked to food availability. This was not measured.
The authors briefly stated at the end of the discussion that the availability of non-native species was not measured. Thus, we cannot say for sure that it contributes to the establishment of Plethodon. But one thing is certain: this species has plasticity regarding its feeding niche, which probably contributed to its status as an invasive species.**

**Second: I'd like to congratulate the authors on the extensive literature review.
However, some of the manuscripts date back to the 1980s and 1990s. That said, the amphibians' diet could have changed over time due to the potential introduction of new non-native prey. It is something to think about.**

**Therefore, I suggest adding a paragraph discussing these limitations. Overall, I've found the manuscript really interesting and enjoyable.**

Thank you for your kind appraisal of our work and for highlighting important considerations regarding the study’s limitations. We agree that prey availability plays a critical role in shaping diet, and we have updated the discussion to more clearly articulate the limitation of not having measured invertebrate abundance or diversity in our system (see Lines: 524-532). While we propose future work to address this gap, we acknowledge that without such data, we cannot definitively determine whether dietary shifts reflect active prey selection or are simply a response to local availability. We hope this paragraph makes the limitation and direction for future work clear to the reader.

Regarding the use of literature from the 1980s and 1990s, we appreciate this observation and agree that there is the potential for prey communities may have changed over time within the native range, potentially due to the introduction of non-native invertebrates. However, given the scarcity of natural history studies, particularly those focused on amphibian diet, we believe it remains valuable to reference these articles. The fact of the matter the specific prey composition within the native range is typically only specified to the Order or Family level, and there is rarely a mention of non-native prey vs native prey. As such, historic or contemporary studies would not often yield the ability to discern comparisons from much of the published work – regardless of decade published. We feel that this is one of the strengths of this paper, and of the value in publishers supporting more natural history focused research moving forward.

We thank you again for your thoughtful feedback and your support of the manuscript.

**Reviewer 1**

**General Comment**

**First and foremost, congratulations on the work and effort invested in its realization and in advancing scientific knowledge regarding these ecological relationships. Moving on to the review, I have provided suggestions for reflection and alteration, applicable to either this study or future investigations.**

Thank you for your time and kind assessment of our work!

**Minor issues**

**(1) The results can inform invasive species management strategies. If the predatory species is predominantly feeding on other invasive species in terms of volume, this could be considered an ecosystem service (if the objective is to control these invasive prey). However, if the priority is to protect native species that are also consumed (even if in smaller volume), removal of the invasive predatory species might be necessary to reduce pressure on native fauna (Simberloff, 2003). Could the study propose management that considers the balance between the direct and indirect negative impacts of the invasive species across all its trophic interactions?**

We agree with Reviewer 1’s comment that our work could inform invasive species management and that this situation lends itself to interesting management question. However, we currently do not have enough ecological information on the system to discuss our findings in terms of this next step, and as such do not feel comfortable making management recommendations within the context of this manuscript. We are happy to report that our work is continuing with these populations, as more work needs to be done before we can understand if this species is providing an ecological service or ecological harm - and how these two facets balance out. We agree this is an interesting and valuable aspect to examine, and we are working with the provincial government wildlife managers to apply what we know to make a plan moving forward.

**(2) Results – When analyzing the consumed volume, there is a highly significant difference, with invasive prey contributing a much larger proportion of the total diet volume (~67.6%) compared to native prey. What explains this?**

This statistical difference is explained by the fact that in general the native prey is significantly smaller than the invasive prey. For example, mites were one of the smallest prey items found in the stomachs of the salamanders but occurred in high numbers (*n* = 189). In comparison, earthworms were some of the largest prey items but there were significantly fewer (*n* = 29) in the diet samples. To convey this to the readers, we have added text to the manuscript to this effect. This text reads “*This disparity in dietary composition based on count and volume is driven by the size differences between prey types, with native invertebrates (e.g. Acari (mites), Collembola (springtails)) being consumed in high numbers but contributing little to overall volume, while larger invasive prey (e.g., Annelida (earthworms)) contribute significantly more despite being less frequent*” (see Lines: 504-508).

**(3) Results – I perceive a statistically very significant difference in the volumes of prey consumed, depending on their status (Native vs. Invasive), by calculating the proportion of the volume. This contrasts with the analysis of percentage by individual, which was not significant. What accounts for this contrast? Please discuss further.**

As discussed above, this difference is explained by the fact that in general the native prey is significantly smaller than the invasive prey. We have added text to the manuscript to this effect. This text reads “*This disparity in dietary composition based on count and volume is driven by the size differences between prey types, with native invertebrates (e.g. Acari (mites), Collembola (springtails) being consumed in high numbers but contributing little to overall volume, while larger invasive prey (e.g., Annelida (earthworms) contribute significantly more despite being less frequent*” (see Lines: 504-508).

**(4) Analysis – Indicator Species Analysis (IndVal) – Identify which prey items are significant indicators of native or invasive populations, or of certain geographical regions. This could reveal specializations or niche shifts.**

We appreciate the Reviewer 1s suggestion to explore Indicator Species Analysis as a method to identify prey items that may serve as significant indicators of native versus invasive populations, or of specific geographical regions. However, we believe that the use of IndVal is limited in our study due to the nature of the dataset and methodological constraints. Due to the use of a literature review in our study the dataset only represents a subset of the invertebrate community. Because of this the data is inherently biased by predator selectivity and does not represent the total community structure, which is a foundational assumption of IndVal. We suggest that future studies that involve standardized field-based diet and environmental sampling in Newfoundland (see lines 524-534). Unfortunately, it is impossible to find the full prey community assemblages from the literature review locations.

**Optional ideas**

**Methods – Although the percentage volume of invasive vs. native prey per individual specimen was not significantly different (p = 0.075), the analysis of aggregated total volume reveals that the consumption of invasive prey has a substantially greater contribution to the total ingested biomass. In summary, despite the absence of clear geographical patterns, the diet of the studied species is strongly influenced by invasive prey in terms of total consumed biomass, which can have significant ecological implications for trophic dynamics and the success of the invasive population. The analyses performed provided important insights but also raised questions that could be further explored in this work and/or for future investigations, given the existing limitations of methods and collected data. Consider the following points if deemed necessary and coherent.**

**(1) Analysis – Multivariate Models for Diet Diversity GLM(M) Multiple – Instead of modeling Shannon diversity with just Lat or Log separately, combine them into a single model: Shannon_Index ~ Lat + Log. Add other environmental variables or population/specimen characteristics that might influence the diet (e.g., individual size, predator sex, habitat type, local temperature, precipitation, altitude). This would help understand the relative contribution of each factor and if they interact. – If this article aims to answer this, clearly state the limitation if it is not achieved.**

We agree with Reviewer 1s comment that combining Lat and Log as well as environmental variables could add more information into the model. Unfortunately, due to the nature of the literature review we do not have information on the environmental or physical characteristics that may influence diet. For example, we do not have the size or sex of individual salamanders for all the studies. We don't have predator data at each site, nor the habitat types/location temperatures/precipitation. This is most notably the case because the year of the studies vary, so we cannot likely get reliable data for all of these. Per Reviewer 1s suggestion we have chosen to add altitude as that is not likely to change over time. To see if the variables could be combined into one model, we completed a Spearman’s rank correlation test. All three variables are significantly corelated. Therefore, we cannot combine them into one model as it could increase the multicollinearity and thus the chances of type two errors. We have included text in the manuscript to this effect, “*As a first step, we tested the correlations between latitudinal, longitudinal, and altitude using the Spearman’s rank correlation coefficient with the function ‘rcorr’ in the ‘Hmisc’ R package [80]. There was a positive correlation between the variables’ latitude and longitude (R2 = 0.84, p < 0.001), a negative correlation between the variables’ latitude and altitude (R2 = -0.30, p < 0.001), as well as a negative correlation between the variables’ longitude and altitude (R2 = -0.17, p < 0.001). As we cannot include correlated variables within the same model, we used separate generalised linear models (GLMs) to test whether prey diversity was related to latitudinal, longitudinal, or altitude gradients using the function ‘glm’ in the ‘glmnet’ R package [81]*.” (see lines 294 to 302).

**(2) Methods – Both models show a positive trend for their respective predictors (Lat and Log). Neither predictor achieves statistical significance at the 0.05 level. The model with Log (g2) has a slightly lower AIC, suggesting it explains slightly more of the variation in Shannon diversity than the model with Lat, although the difference is minimal and significance is still marginal. This indicates that neither latitude nor longitude, individually, are strong predictors of diet diversity in these univariate analyses, although there is a suggestion that diversity may increase at higher latitudes and longitudes. It would be interesting to explore a model that included both variables or others, or consider the interaction between them, to see if a combination better explains the variation in diet diversity. Subsequently, the results suggest that both latitude and longitude have a limited and statistically non-significant influence (at the standard confidence level) on individual diet diversity. Although there is a trend of increasing diversity with higher latitudes and longitudes, this is not robust enough to be considered a clear and predictive relationship based on this dataset. This might indicate that other factors not included in the models (climatic, environmental, biological) or the complexity of geospatial interactions may be more important, or that the sample size is insufficient to detect more subtle effects (just make this clear in the text, limitations are fine). This suggests a trend of increasing diet diversity with longitude, but the evidence is not strong enough to reject the null hypothesis (that there is no relationship) – A question to be posed for long-term future study investigations.**

As discussed above, we could not combine latitude and longitude into the same model because of the correlation between the two variables (*R*^2^ = 0.84, *p* < 0.001). We have made this clear in lines 294 to 302.

**Reviewer #2**

**General Comment**

**This paper provided an excellent overview of multiple invasion theories and prior literature. It was well set up to neatly test the authors questions. The methods and stats are all sound and I only had a few issues to raise in addition to some line by line comments. I believe once rectified, the paper will be suitable for publication.**

We very much appreciate Reviewer 2’s kind assessment of our manuscript. Thank you!

**1. The introduction (whilst very informative) is somewhat clunky in places. There are some line by line comments below, but I found that overall, many of the sentences were too long and thus felt like ‘information overload’. Please go over the introduction and break up some of the sentences, plus remove word ‘double ups’ in the same or close by sentences.**

We appreciate the detailed recommendations Reviewer 2 has made regarding the clunky sentences and word ‘double-ups’ below. We have gone through the supplied PDF and have made many of the suggested edits across the manuscript.

**2. The authors did not make direct comparisons between the invasive and native range population in terms of how much invasive prey they consumed. As far as I can tell – only the invasive population’s diet was examined in this way. But that’s an important part of the interpretation. If native range population eat equal amounts of invasive prey then that changes the story. Please include more results and subsequent discussion of this aspect directly.**

We appreciate this point brought up by Reviewer 2. Unfortunately, our field work was only completed on the invasive population. Our literature review compiled the general diet of *Plethodon cinereus* in their native range, covered 14 studies and 24 populations, however the degree of taxonomic resolution for most of these studies was only as detailed to the Order or Family level, and the authors did not distinguish between invasive/native prey. In general, most of the studies that were used in the literature analysis did not use specific enough levels of prey identification to identify if the prey was an invasive/native species. Therefore, we were unable to categorise and back-calculate the native or invasive status of each prey items from the literature review. One study from which we compiled data mentioned invasive prey items (see Scott et al, 2024). However, this study only examined earthworms as invasive prey items and did not consider that other prey items may have been invasive as well.

We very much agree that such an examination would allow for more in-depth examinations of the diet – yielding strong data for comparison between invasive and native populations. It is our hope that our manuscript will prompt more detail natural history data collect, allowing for the comparison the Reviewer has suggested to be done in the future. We have included text in the manuscript to this effect (see Lines: 526-532).

**3. A little more explanation is required regarding the invasive population status – how are the authors certain that these populations are invasive? Is a delay in reporting animals on the island merely because there was less scrutiny of the biodiversity previously? Do island inhabitants remember the invasion arriving etc. It seems unusual that these animals wouldn’t be found on close by islands, but perhaps not for someone that knows the system better.**

We appreciate the reviewer’s request for clarification regarding the invasive status of the *Plethodon cinereus* population in Newfoundland. There are no native reptiles or amphibians on the island of Newfoundland. Large scale biological surveys have been completed on the islands and began in 1877. The first confirmed record of *P. cinereus* in Newfoundland was made by Baxter-Gilbert et al. 2022 (see Lines 140-142). Moreover, the nearest native populations of *P. cinereus* are approximately 517 kilometers from the island of Newfoundland, in Nova Scotia. Therefore, it is unlikely that the salamanders or any other herpetofauna could have come through natural circumstances. Our research group is currently drafting a molecular ecology manuscript that details the origin of invasive Newfoundland *P. cinereus*, where we have compared the genetics of the Newfoundland population to those from across eastern Canada. This work has demonstrated the genetic origins links tightly to Cape Breaton, and nowhere else in the Maritimes. This work is expected to be submitted early next year. We have now added text to better explain that this island was devoid of amphibians previous to human intervention (see Lines: 137-140).

**Specific comments**

**Line 37: ‘bolstered’?**

Thank you for catching this typo. We have change ‘bolster’ to ‘bolstered’ (see Line: 37).

**Line 49: too close to use ‘across the globe’ again.**

Thank you for this suggestion, we completely agree. We have changed the second mention of ‘globe’ to “… over 40 countries…” (see Lines: 49-53).

**Lines 58-59: clunky use of ‘increase’ twice – reword.**

We have reworded the first instance of the word “increase” to “rise” (see Line: 59-61).

**Lines 60-61: double use of expanding in one sentence**

Thank you for this suggestion. To avoid the repetition, we have reworded the second use of “expanding” to “growing” (see Line: 61-63).

**Line 73: reword ‘their introductions into Mauritius and Tanzania both failed’.**

Thank you for your suggestion. We have updated the sentence to be more concise. “For example, although the Cane Toad is one of the most significant vertebrate invaders … their introductions into Mauritius and Tanzania both failed” (see Lines: 74-76).

**Lines 78 – 86: great overview!**

Thank you for your kind assessment.

**Lines 91-92: sentence to convoluted – divide.**

Thank you for the suggestion. We agree – this one had some twists and turns. We have now scaled back and rewritten this sentence to be much clearer. The text now reads: “*The value of a broad dietary niche promoting invasion success is linked to the concept that if a colonizing population can more widely diversify what they eat, then they can minimise the degree of direct competition with specific native taxa and thus increasing their invasive potential (i.e., Niche Breadth Invasion Success Hypothesis [23]).*” (see Lines: 94-97).

**Line 152: compared ‘it’? be careful with your wording – I would change the whole sentence to be clearer: e.g. To do investigate this, we compared the diets of invasive vs. non-invasive populations of salamanders. We analysed stomach contents form the invasive populations and did systematic literature reviews to characterise the diet of the species in its natural range.**

Thank you for this comment. We agree, ‘it’ can be quite vague. We have reworded the sentence to be clearer (see Lines: 154-157). The text now reads: “*To investigate this, we analyzed the stomach contents of salamanders collected from Newfoundland and compared these results to a dataset of this species’ diet across its native range that we generated from a systematic literature review.*”

**Line 177: do you know how or why they got there?**

At this point in time, we unfortunately do not know how or why the salamanders arrived in Newfoundland. Currently work is being done to answer this question, and work we have conducted and are in the process of drafting into a manuscript suggests they came from Cape Breaton. Once we are sure of the genetic source population, it is our hope that we will be able to elucidate the invasion pathway (and maybe even timing) of their arrival.

**Line 195: how do you sex them?**

To increase clarity, we have updated our sentence to include more information on our sexing techniques as well as added a citation for teste candling (see Lines: 197-200). The text now reads “*When an individual was found, it was captured by hand and sexed using secondary sex characteristics and candling to determine whether testes where present [54]. Age was assessed (adult or juvenile) by length, the adult threshold was a snout to posterior cloaca of at least 32 mm [55]. Salamanders were then humanely euthanized in a MS-222 (tricaine mesylate) bath, containing 500 mg/L of MS-222 (buffered with sodium bicarbonate to a pH ~7).”*

**Line 221: could some species be missed in the literature because they are digested prior to analysis?**

Reviewer 2 makes an excellent point as many prey items, especially soft-bodied items were highly digested and could not be identified. These prey items were labeled as unknown in our analysis. To increase clarity, we now have now added this concept into the paragraph on limitations in the Discussion (see line 535-538). The text now reads: “*Additionally, soft-bodied prey items are less likely to be uncovered in diet analysis studies due to their level of digestion and loss of identifiable characteristics. By preforming a molecular analysis of the dietary samples, a higher level of specificity could be achieved for highly digested or incomplete prey items*.”

**Line 239: has this species name stayed consistent since 1967?**

Yes. *Plethodon cinereus* has been the species scientific name for Eastern Red-backed Salamanders since 1818.

**Line 282: ‘how frequently’?**

Thank you to Reviewer 2 for catching this typo. We have updated “frequent” to “frequently” (see Line: 289).

**328 and 338, 387 and 388, 392 etc.: include common names also where possible (e.g. ‘mites’, ‘beetles’, ‘nematodes’) as it keeps the text more engaging.**

We appreciate Reviewer 2’s suggestion. Although we do not fully agree that using the proper names for taxonomic group is unengaging, we have included the common names for the groups more frequently across the manuscript for readers that are less familiar with them. We can see common names for taxonomic groups in Table 1, and in our discussion of the diet composition breakdown in the Results (see Lines 342-346, 361-367, and Table 1) and in the Discussion (see Lines 461-464, 505-509, and 539-541).

**Line 405: was there any invasive prey in the native range diets? I can’t see that mentioned anywhere but that seems important.**

Thank you. This is a really good point, and it is one that we grappled with during our study, and one that Reviewer 1 bought up as well. Please see our response above to Reviewer ‘s comment #2 for details.

**Line 447: interesting!**

Thank you for your kind assessment.

**Line 507: I would have thought this would be relatively easy to do from literature already?**

We agree this is an interesting point (i.e., regarding the caloric value of taxa). However, the dietary value (and range) for different taxa may have some larger taxonomic trend for prey items, however this will understandably vary widely across the geographic range of a prey species and from population to population. Although, this information may exist for certain prey species across their range, there are no studies conducted at the CSB location in Newfoundland. As such, whatever nutritional data we were able to find on these prey items would be viewed as speculative at best – and thus we were hesitant to include such data in this manuscript. That said, this is a question we discussed at length, and see the value of this added layer of insight. After reflection on this we felt that it would be better to suggest future work (which our lab may undertake) collect samples from the known prey groups at this site specifically. That is, we feel comparisons of proverbial ‘apples to apples’ is a stronger and more appropriate approach compared to a more assumption-based presentation at this point in this research.
