# Supplementary material for: In the belly of the beast: How dietary changes and preexisting invasive prey may have promoted the success of a novel invasive amphibian
Source: PLoS One. 2026 Jul 10;21(7):e0352970. doi: 10.1371/journal.pone.0352970 (PMC13354068; doi:10.1371/journal.pone.0352970)
Supplement: Attachment — Submitted filename: Review-eng-mau-02.docx [file pone.0352970.s003.docx]

Dear authors,

Firstly, congratulations on your work and efforts in making the corrections.

(1) Methods - I would have liked to see a map with scales and a better location of where the study was conducted, the geographical coverage it achieved, if possible, to make this visual identification.

Example:

Source: <https://doi.org/10.22201/ib.20078706e.2022.93.4130> by Cid-Muñoz et al. 2022.
