# Supplementary material for: In the belly of the beast: How dietary changes and preexisting invasive prey may have promoted the success of a novel invasive amphibian
Source: PLoS One. 2026 Jul 10;21(7):e0352970. doi: 10.1371/journal.pone.0352970 (PMC13354068; doi:10.1371/journal.pone.0352970)
Supplement: Attachment — Submitted filename: PONE-D-25-22281R1.docx [file pone.0352970.s004.docx]

Manuscript number – PONE-D-25-22281R1

In the Belly of the Beast: How Dietary Changes and Preexisting Invasive Prey may

have Promoted the Success of a Novel Invasive Amphibian

This paper reports the stomach contents of a robust sample of the population of Eastern Red-backed Salamanders on Newfoundland and attempts to make comparisons to the existing diet data for this species from its native range. The paper is well written but, in my view, needs to be recast in the context of optimal foraging and natural history of the species. There are some good data here but the comparison to diets from the chosen literature doesn’t work and the interpretation of the results is very much overwritten.

I will outline the general issues and provide some more specific ideas for improvement.

The introduction of the study is based on testing two hypotheses (the Niche Breadth Invasion Success Hypothesis and the Invasional Meltdown Hypothesis). To test the first hypothesis the authors predicted that the non-native population of salamanders would have higher species richness, a higher diversity index score and greater evenness of prey types compared to native populations. There are flaws in these data that I will detail below but essentially the available diet data extracted from 14 papers published on diets in the native range are presented in different ways in the literature and so the derivation of the diversity indices is not comparable to the author’s data set. See specific examples below.

The authors then predicted that the non-native population would have greater than 30% (by count and volume) non-native prey if the Invasional Meltdown Hypothesis is supported. The 30 % is somewhat arbitrary (midway between ranges reported in the literature of 3-55% by count and 16-71% by volume. For example, if the authors chose 40% cut-off instead, they would interpret their data slightly differently and their chi-square analyses would be based on a different arbitrary expected value. An additional problem here is that red-backed salamanders in their native range are known to be generalist predators that feed on a variety of native AND non-native prey types, so it isn’t as though they only feed on exotic prey in Newfoundland where they were introduced apparently recently. The citations below have some data on non-native prey in the diet elsewhere.

Maerz JC, Karuzas JM, Madison DM, Blossey B (2005) Introduced invertebrates are important prey for a generalist predator. Diversity Distrib 11:83–90

Walton BM, Tsatiris D, Rivera-Sostre M (2006) Salamanders in forest-floor food webs: invertebrate species composition influences top-down effects. Pedobiologia 50:313–321

Ivanov K, Lockhart OM, Keiper J, Walton BM. Status of the exotic ant *Nylanderia flavipes* (Hymenoptera: Formicidae) in northeastern Ohio. Biol Invasions. 2011;13:1945-50. doi: 10.1007/s10530-011-0021-z

Discussion in: Paluh, Daniel J., et al. "Selective foraging on ants by a terrestrial polymorphic salamander." *The American Midland Naturalist* 174.2 (2015): 265-277.

I don’t think that the comparisons made are a good test of either hypothesis set up in the intro.

**Line by line/section comments**

Since I think the paper needs to be reworked without the two hypotheses, I skipped looking for typos in most parts of the paper, but I tried to comment on things that I thought were particularly problematic. Also, in my opinion the word “invasive” is misused throughout the paper. Invasive species/population are defined as “those introduced to a new ecosystem, often by humans, that spread aggressively and cause significant economic, environmental, or ecological harm by outcompeting natives, damaging habitats, reducing biodiversity, and disrupting ecosystem functions” Do we know if cinereus is spreading in Newfoundland? Do we know if *cinereus* there are having negative ecological effects or negative effects on the economy? If so, cite some literature to back up that they are invasive. Otherwise consider using another term to describe your population (e.g. non-native, exotic, introduced). Especially because native species can be invasive in some cases.

Line 168: lower case p in plethodontid

Line 192: should be Red-backed. Check throughout

Lines 198-200: Did the diets of males and females differ? Juveniles and adults?

Line 219: There is no way to measure small prey items like mites and springtails with calipers. Is this a mistake?

Line 252: salamanders plural

Lines 256-259: I do not think that this approach is valid because it likely makes the diversity indices incomparable. Please explain exactly how the index was calculated for your own data and for the data extracted from the literature. For example, you could (and should), at least for your own data, calculate a diversity index for each individual animal’s stomach contents. This of course allows you to compare means statistically. Obviously, this isn’t doable for most of the published literature without having the raw data so how did you deal with it? You need to explain exactly what you did because some papers report mean diversity based on calculations of diversity per individual (Anthony et al. 2008 and Hantak et al. 2020 and Stuczka et al 2016) other papers do this differently by providing only an index for a sample.

I did not go to each of the 14 papers used in this study but there were issues with the ones I did examine which makes me skeptical that the others were handled properly.

For example, it is unclear how the authors arrived at H’ = 1.96 for the Anthony et al data. That paper does not provide raw data, and the mean H’ for the striped morph = 2.71 and unstriped = 2.39.

The supplemental data in Hantak et al provides a list of prey species and total numbers of each of those species/morphospecies in the study. Fig. 3 in that paper reports 8 indices for 6 localities and 2 morphs (in two seasons if the supplemental are included). Diversity indices could either be calculated from the list of prey, or extracted from Fig. 3 + or - the supplemental fig. The results would differ depending on which approach was used. So how was H’ derived in S2 for this paper?

The sample size reported in Table S2 for the Raimondo et al 2003 paper is not correct. It should be 56 not 263.

Additionally, this paper only examined the 7 most common prey categories which is inappropriate to use for calculating H’, evenness and richness.

Also, it is problematic to include data with unknown sample size and those natural history notes with n=1. If you look at the relationship between sample size and H’ you find a positive one. Studies that examined more salamander stomach contents generally have higher diversity scores. This is true if you exclude the unknown sample size, the n=1s, and fix the sample size for the Raimondo et al paper. Furthermore, the natural history notes should be excluded because they tend to be unrepresentative examples of the diet. For example, one of the used notes documents *cinereus* diet from a carnivorous plant in a bog so of course that individual’s gut was filled with flies.

Unfortunately, these problems make it difficult to trust the remaining diversity data and so the niche breadth comparison doesn’t seem valid, especially because diversity indices could have been calculated differently and even more importantly, diversity scores are dependent on sample size.

Line 287: Table S3 is confusing. The columns %N and %I seem like they should be combined in a column with Y or N. As is, the columns are all either 100% or 0%

Line 306: “compared” is missing before “to conspecifics”

Line 354 Table 1: Collembola are missing

To me it is strange that all comparisons differ statistically in this table. What is the biological relevance of a difference of for example of 0.005% versus 0. What are the expected values for these tests and how are you interpreting these results?

Also from the table it appears as though species richness is greater in the native range but of course the sample size collectively is much bigger for the combined literature and you likely cannot report a mean richness for the native data.

Line 357 Table 2: I think if you carefully look at the literature on diets from the native range you will see that by number mites and Collembola make up the majority of the diet which mirrors your findings, except interestingly you found many beetles. Were those beetles larvae or adults?

Another interesting finding is that you found quite a few centipedes in your stomachs which by volume is significant and unique compared to the native range diets. Isopods are a common prey item in the native range as well. Check out the importance values in the literature that you used to extract diet data. Most of those are exotic species of isopods.

Mesostigmata appears in Table 2 twice

Line 392: Table 3 does not give any additional data that are not in the text so I would get rid of it even if you can argue the validity of the info in Table S2.

Lines 441-449: I think this paragraph is overwritten and too strongly worded. Without data on diet preferences which would require samples of the available prey you cannot say that the non-native population has shifted its dietary niche to non-native prey. BY number your samples still have mostly mites and springtails. In the native range this species is truly a generalist predator and for the most part they prey on whatever is in abundance (native or non-native prey) when foraging conditions are less than ideal (warm or dry). When conditions are cool and wet foraging occurs more broadly, and salamanders selectively consume prey that are soft bodied and pass through the gut quickly (i.e. less chitinous). We do know that *cinereus* can and do avoid certain available prey and select certain other prey (see Paluh et al 2015 which examines selective foraging on ants by striped and unstriped morphs of cinereus). Maybe *cinereus* in Newfoundland are selectively foraging on larger, chitinous prey that happen to be exotic, or maybe those are just the prey that were most available and offered the biggest for there buck when you sampled, but without knowing what prey taxa are available it would be difficult to say and certainly. you cannot claim a shift in diet from the native range without a clearer comparison to data that were collected and analyzed the same way. So minimally the writing must be toned down.

Lines 478-484: I don’t think you can say that differences in the diets reported in this study represent real differences. This would be an assumption if we knew that the available prey communities were relatively the same in both native and non-native ranges, but we do not have that information. Richness was greater in the native range (Table 1) and the diversity indices are flawed so the statements in this part of the discussion are not supported by the data. Additionally, we know from papers published by both BM Walton and RG Jaeger that litter invertebrate prey populations fluctuate drastically by season primarily due to litter thickness and moisture. Such fluctuations result in seasonal differences in competition for prey. Competition weakens in late spring and fall when resources, and good foraging conditions, are abundant. In the summer and early fall competition for limited or lower quality resources resumes resulting in partitioning of the prey base in ways that territorial residents monopolize the best foraging areas while floaters are only able to acquire lower quality prey (those that are heavily armored and pass through the gut more slowly). This is another reason that comparing your data to combined data from other studies is problematic. I am sure data were collected from all seasons in the native range dataset whereas these data were collected in May and June.

Bibliography on supplement – The Hughes citation was in issue 3, not 2

My review is not meant to be negative. I think there are good diet data here on a population of red-backed salamanders that is introduced to Newfoundland which we know little about. My recommendation would be to focus on the non-native data and discuss them in the context of what is known about foraging tactics in this species.

Jaeger 1990 and citation therein would be a great place to start. You may be able to add a quick experiment to your diet data to test some of the hypotheses laid out in this paper.

Jaeger, Robert G. "Territorial salamanders evaluate size and chitinous content of arthropod prey." In *Behavioural mechanisms of food selection*, pp. 111-126. Berlin, Heidelberg: Springer Berlin Heidelberg, 1990.

another helpful paper

Daniel J. Paluh, Cameron Eddy, Kaloyan Ivanov, Cari-Ann M. Hickerson, and Carl D. Anthony "Selective Foraging on Ants by a Terrestrial Polymorphic Salamander," *The American Midland Naturalist* 174(2), 265-277, (1 October 2015). <https://doi.org/10.1674/0003-0031-174.2.265>

Alternatively, if you choose to keep the focus on the extracted data from the literature, I would make certain that the data are comparable which likely means excluding some of the papers and acquiring raw data from authors to ensure that the analyses and calculations are the same and done correctly. This would include ensuring similar sample sizes from native and non-native ranges with data collected at the same time of year and at similar elevation.
