# Supplementary material for: In the belly of the beast: How dietary changes and preexisting invasive prey may have promoted the success of a novel invasive amphibian
Source: PLoS One. 2026 Jul 10;21(7):e0352970. doi: 10.1371/journal.pone.0352970 (PMC13354068; doi:10.1371/journal.pone.0352970)
Supplement: Attachment — Submitted filename: Response to Reviewers v5_Williams et al.docx [file pone.0352970.s005.docx]

**Responses to Reviewers**

*Comments are in bold with the responses in plain text immediately below.*

**Academic Editor Comments**

**Journal Requirements:**

**1. If the reviewer comments include a recommendation to cite specific previously published works, please review and evaluate these publications to determine whether they are relevant and should be cited. There is no requirement to cite these works unless the editor has indicated otherwise.**

Thank you for this note, we will do so. In particular, the Maerz et al. 2005 citation makes an excellent point about native range salamander also feeding on invasive prey. This fact strengthens are contextualisation as to why we choose to look at diet, and how this flexibility could have allows for the salamanders to succeed under an Invasional Meltdown Hypothesis framework. We appreciate Reviewer 3 bringing this to our attention and have included it in the text (see Lines: 143-146).

**2. Please review your reference list to ensure that it is complete and correct. If you have cited papers that have been retracted, please include the rationale for doing so in the manuscript text, or remove these references and replace them with relevant current references. Any changes to the reference list should be mentioned in the rebuttal letter that accompanies your revised manuscript. If you need to cite a retracted article, indicate the article’s retracted status in the References list and also include a citation and full reference for the retraction notice.**

Thank you. We have gone through all our cited literature and our literature review list. There were no papers that were retracted. If you are aware of a specific instance and we have missed it, please let us know.

**[Note: HTML markup is below. Please do not edit.]**

As instructed, we have not responded directly to the comments made within this section.

**Reviewers' Comments**

**Reviewer's Responses to Questions**

**Comments to the Author**

**1. If the authors have adequately addressed your comments raised in a previous round of review and you feel that this manuscript is now acceptable for publication, you may indicate that here to bypass the “Comments to the Author” section, enter your conflict of interest statement in the “Confidential to Editor” section, and submit your "Accept" recommendation.**

**Reviewer #1: All comments have been addressed**

**Reviewer #3: (No Response)**

**2. Is the manuscript technically sound, and do the data support the conclusions? The manuscript must describe a technically sound piece of scientific research with data that supports the conclusions. Experiments must have been conducted rigorously, with appropriate controls, replication, and sample sizes. The conclusions must be drawn appropriately based on the data presented.**

**Reviewer #1: Yes**

**Reviewer #3: No**

**3. Has the statistical analysis been performed appropriately and rigorously?**

**Reviewer #1: Yes**

**Reviewer #3: I Don't Know**

**4. Have the authors made all data underlying the findings in their manuscript fully available? The** [**PLOS Data policy**](http://track.editorialmanager.com/CL0/http:%2F%2Fwww.plosone.org%2Fstatic%2Fpolicies.action%23sharing/1/010f019b0073e2a5-a44bfef1-f484-4407-88c5-f0d0262bb1d0-000000/jTLPTlytWygT-yhe1b-LkdvqyJFnV_SG3AE3rCLISdI=240) **requires authors to make all data underlying the findings described in their manuscript fully available without restriction, with rare exception (please refer to the Data Availability Statement in the manuscript PDF file). The data should be provided as part of the manuscript or its supporting information, or deposited to a public repository. For example, in addition to summary statistics, the data points behind means, medians and variance measures should be available. If there are restrictions on publicly sharing data—e.g. participant privacy or use of data from a third party—those must be specified.**

**Reviewer #1: Yes**

**Reviewer #3: Yes**

**5. Is the manuscript presented in an intelligible fashion and written in standard English? PLOS ONE does not copyedit accepted manuscripts, so the language in submitted articles must be clear, correct, and unambiguous. Any typographical or grammatical errors should be corrected at revision, so please note any specific errors here.**

**Reviewer #1: Yes**

**Reviewer #3: Yes**

**6. Review Comments to the Author. Please use the space provided to explain your answers to the questions above. You may also include additional comments for the author, including concerns about dual publication, research ethics, or publication ethics. (Please upload your review as an attachment if it exceeds 20,000 characters)**

**Reviewer #1: The answers provided were satisfactory. There are changes to be made, but overall, questions were answered and clarified.**

**Reviewer #3 (uploaded as a separate file): This paper reports the stomach contents of a robust sample of the population of Eastern Red-backed Salamanders on Newfoundland and attempts to make comparisons to the existing diet data for this species from its native range. The paper is well written but, in my view, needs to be recast in the context of optimal foraging and natural history of the species. The niche breadth analysis is not correct and the interpretation of the data is an overreach.**

**7. PLOS authors have the option to publish the peer review history of their article (**[**what does this mean?**](https://track.editorialmanager.com/CL0/https:%2F%2Fjournals.plos.org%2Fplosone%2Fs%2Feditorial-and-peer-review-process%23loc-peer-review-history/1/010f019b0073e2a5-a44bfef1-f484-4407-88c5-f0d0262bb1d0-000000/-p5uz-i3wbjD0JpekutCaL-tq8QLu7Ay0vsSJ1gcDuA=240)**). If published, this will include your full peer review and any attached files. If you choose “no”, your identity will remain anonymous but your review may still be made public. Do you want your identity to be public for this peer review? For information about this choice, including consent withdrawal, please see our** [**Privacy Policy**](https://track.editorialmanager.com/CL0/https:%2F%2Fwww.plos.org%2Fprivacy-policy/1/010f019b0073e2a5-a44bfef1-f484-4407-88c5-f0d0262bb1d0-000000/9oC6yAYyoCxgg94zkAANzB7XpQNt8SAiwlrIXlgKS5s=240)**.**

**Reviewer #1: Yes: Marcos Akira-Umeno**

**Reviewer #3: No**

**Reviewer 1**

**General Comment**

**Firstly, congratulations on your work and efforts in making the corrections.**

(1) **Methods - I would have liked to see a map with scales and a better location of where the study was conducted, the geographical coverage it achieved, if possible, to make this visual identification.**

**Example:**

**Source:** [**https://doi.org/10.22201/ib.20078706e.2022.93.4130**](https://doi.org/10.22201/ib.20078706e.2022.93.4130) **by Cid-Muñoz et al. 2022.**

Thank you for your comment on improving our map visualisation. We have added a scale bar and north arrow for clarity (see below and within the resubmission). The geographic coverage was taken from *IUCN’s Red List (International Union for Conservation of Nature. 2022. Plethodon cinereus. The IUCN Red List of Threatened Species. Version 2017-3. https://www.iucnredlist.org. Downloaded on 18 December 2023*) and we have included this reference in the figure caption (see Line 355).

**Reviewer 3**

**Dear authors,**

**Manuscript number – PONE-D-25-22281R1**

**In the Belly of the Beast: How Dietary Changes and Preexisting Invasive Prey may**

**have Promoted the Success of a Novel Invasive Amphibian**

**This paper reports the stomach contents of a robust sample of the population of Eastern Red-backed Salamanders on Newfoundland and attempts to make comparisons to the existing diet data for this species from its native range. The paper is well written but, in my view, needs to be recast in the context of optimal foraging and natural history of the species. There are some good data here but the comparison to diets from the chosen literature doesn’t work and the interpretation of the results is very much overwritten.**

We appreciate Reviewer 3’s perspective on how they would choose to frame this study (i.e., as an optimal foraging and natural history account), however we do not agree with their assertion that our data does not allow for the comparisons and interpretations we have presented here. First and foremost, we do not have the appropriate data to test for optimum foraging (e.g., nutrient/energy yield of prey, nor prey electivity) and, owing to the comparative nature of our work, we would assert that this is far more than a simple description of natural history. We are pleased to see that both Reviewer 1 and 2 agree that our approach to test our selected invasion hypotheses was correct and appropriate. Overall, we can see how Reviewer 3 may personally lean toward a more optimum foraging and natural history framework, but we do not think that it would be suitable for this work, and we assert the approach we have taken is valid. Below we will outline why we feel this way, and add additional text in the main text of the manuscript to better clarify and contextualise this point. We hope these differences in opinion will be acceptable.

**I will outline the general issues and provide some more specific ideas for improvement.**

**The introduction of the study is based on testing two hypotheses (the Niche Breadth Invasion Success Hypothesis and the Invasional Meltdown Hypothesis). To test the first hypothesis the authors predicted that the non-native population of salamanders would have higher species richness, a higher diversity index score and greater evenness of prey types compared to native populations. There are flaws in these data that I will detail below but essentially the available diet data extracted from 14 papers published on diets in the native range are presented in different ways in the literature and so the derivation of the diversity indices is not comparable to the author’s data set. See specific examples below.**

**The authors then predicted that the non-native population would have greater than 30% (by count and volume) non-native prey if the** **Invasional Meltdown Hypothesis is supported. The 30 % is somewhat arbitrary (midway between ranges reported in the literature of 3-55% by count and 16-71% by volume. For example, if the authors chose 40% cut-off instead, they would interpret their data slightly differently and their chi-square analyses would be based on a different arbitrary expected value.**

Reviewer 3 brings up a sound point, and this was a topic that we sat with and contemplated for a good deal of time when we were designing this experiment. The fact of the matter is, within the literature there is no hardline or threshold by which dietary contributions within an ‘Invasional Meltdown’ context would be considered to support the hypothesis. Overall, the core concept of the Invasional Meltdown Hypothesis is that the previous establishment of other invasive species provides an advantage to an invading population. As such, it has been suggested this advantage could be gained with an addition of relatively low values, like the 3% and 16% we report and which was noted by Reviewer 3, all the way to the bulk of the diet being non-native prey (55% and 71%). Many of these studies are not comparative between native and invasive predator populations, however, and thus we needed to devise a threshold. To achieve this, we searched for literature reporting invasion success related to non-native diets, and found authors crediting the hypothesis for promoting invasion success from values as low and 3-16% and as high as 55-71%. The mean value for count data was 26% and 27.5% for volume data. Given that our goal was to select a meaningful value by which we could be confident that the dietary contribution by non-native prey was in fact a benefit, we felt like the selection of 30% for both count and volume data in our study would be both appropriate and slightly more conservative. As such, the 30% cut-off was not arbitrary, but rather based on what has been published on this topic previously and what we rationalised as a significant proportion of the diet. (see Lines 329- 334). We could, if the editor prefers, use the 26% and 27.5% calculated median values from the range. However, this would not change the outcome of the study. As such, we assert that our cut-off values are grounded in previous literature and represent a notable proportion of a diet, and thus suitably test of Invasional Meltdown Hypothesis.

**An additional problem here is that red-backed salamanders in their native range are known to be generalist predators that feed on a variety of native AND non-native prey types, so it isn’t as though they only feed on exotic prey in Newfoundland where they were introduced apparently recently. The citations below have some data on non-native prey in the diet elsewhere.**

**Maerz JC, Karuzas JM, Madison DM, Blossey B (2005) Introduced invertebrates are important prey for a generalist predator. Diversity Distrib 11:83–90**

**Walton BM, Tsatiris D, Rivera-Sostre M (2006) Salamanders in forest-floor food webs: invertebrate species composition influences top-down effects. Pedobiologia 50:313–321**

**Ivanov K, Lockhart OM, Keiper J, Walton BM. Status of the exotic ant *Nylanderia flavipes* (Hymenoptera: Formicidae) in northeastern Ohio. Biol Invasions. 2011;13:1945-50. doi: 10.1007/s10530-011-0021-z**

**Discussion in: Paluh, Daniel J., et al. "Selective foraging on ants by a terrestrial polymorphic salamander." *The American Midland Naturalist* 174.2 (2015): 265-277.**

Thank you for these suggestions, we have included the capacity for native red-backed salamander populations to adjust to invasive prey in the manuscript now, and suggest it may be a trait that further bolsters their invasive potential within a dietary Invasional Meltdown Hypothesis framework. The text now reads “*To date, little is known about the invasion history or ecology of this extralimital salamander population, however in their native range this species is known to be a generalist predator that will feed on invasive prey when ecological conditions are favorable [43] – suggesting that dietary flexibility may be a trait that could promote invasion success*.” (see Lines 143-146).

**I don’t think that the comparisons made are a good test of either hypothesis set up in the intro.**

Here we see where some of the confusion about our work, and how the Invasional Meltdown Hypothesis can be applied to dietary studies, may be presenting an issue with Reviewer 3. Once again, we appreciate the Reviewer’s expertise on this species and their feeding habits, and they are quite right that this species is known to eat invasive/non-native prey in their native range. We have now made this point clear in the manuscript and would thank Reviewer 3 for flagging this missing part of the story (see Lines: 143-146).

Nevertheless, the fact that this species has the capacity to eat invasive/non-native prey in their native range when conditions would make it more advantageous does not refute or diminish the Invasional Meltdown Hypothesis framework we are testing in our study. Quite the contrary, it strengthens it. The fact that this salamander is able to shift prey groups when ecological conditions are more favourable to do so (such as that presented in Maerz et al. 2005; which Reviewer 3 kindly flagged) means that they are more likely to use this as a mechanism to minimise the likelihood of competitive exclusion, and capitalise on prey that is underutilised in the invaded range (see Lines: 143-146). Understanding how capitalising on invasive/non-native prey within an Invasional Meltdown Hypothesis framework does not require the invading predator to suddenly take up never before eaten prey types. It simply requires them to be able to use prey that might not come with the same competitive cost as feeding on native prey that already has long evolved relationship with native predators (i.e., the invading predator’s competitor).

Within this study we found that the invasive salamanders of Newfoundland, both broaden their dietary niche (which would begin to reduce competition with native dietary specialists), and then further feed heavily on invasive/non-native prey (which would further reduce competition with native). These two facts can both be true, even with native Eastern Red-backed Salamanders also feeding on invasive/non-native prey when conditions are favourable. Species with this dietary ability are more likely to be more successful invaders. This fact we already present in the manuscript (see Lines: 143-146).

**Specific comments**

**Line by line/section comments**

**Since I think the paper needs to be reworked without the two hypotheses, I skipped looking for typos in most parts of the paper, but I tried to comment on things that I thought were particularly problematic. Also, in my opinion the word “invasive” is misused throughout the paper. Invasive species/population are defined as “those introduced to a new ecosystem, often by humans, that spread aggressively and cause significant economic, environmental, or ecological harm by outcompeting natives, damaging habitats, reducing biodiversity, and disrupting ecosystem functions” Do we know if cinereus is spreading in Newfoundland? Do we know if *cinereus* there are having negative ecological effects or negative effects on the economy? If so, cite some literature to back up that they are invasive. Otherwise consider using another term to describe your population (e.g. non-native, exotic, introduced). Especially because native species can be invasive in some cases.**

We appreciate the reviewer’s request for clarification regarding the invasive status of the *Plethodon cinereus* population in Newfoundland. Firstly, we need to address the definition of ‘invasive’ that was provided. Reviewer 3 uses a more traditional (and possibly regionally legal) definition of the term – in particular the caveat regarding known negative ecological effects. We agree making this definition clear is important. Over time there has been a great deal of debate and discussion on how to define invasive species, which little agreement reached and definition vary regionally and contextually. Within our study, and to prevent confusion, we provide a more contemporary definition early in the manuscript so that the readers are aware of our framing (see Lines 67-70). There we state that “*Invasive populations can form after non-native individuals progress through a series of ‘invasion stages’, whereby they must be able to survive transport to a new location, survive within the new habitat, reproduce and maintain a self-sustaining population, and expand their range beyond the site of initial establishment* [14, 16].”. This definition is based on Richardson et al. (2000) and Blackburn et al. (2011).

With respect to this invasive population in particular, the island of Newfoundland has no native reptiles or amphibians. Large scale biological surveys have been completed on the island, beginning as early as 1877. Yet, the first confirmed record of *P. cinereus* in Newfoundland was made by Baxter-Gilbert et al. 2022 (see Lines 140-142). Moreover, the nearest native populations of *P. cinereus* are approximately 517 kilometers from the island of Newfoundland, in Cape Breton, Nova Scotia. Therefore, it is unlikely that the salamanders or any other herpetofauna could have come through natural circumstances. To date, we have documented the population at the CBS site, and have photographic evidence of individual on both the west and east coast of the island (suggesting within island geographic spread). Furthermore, we have also being provided firsthand account of a nearby population ~20 km the CBS site that was thriving in the mid-1970s. It is unknown if the population is causing an ecological or economic harm. Overall, we are certain that this species has spread within the island from an unknown establishment site; whether the CBS site was the point of introduction or not remains unknown, but also inconsequential give the recent reports. In addition, our lab is currently conducting undertaking a genetic mapping exercise to determine the origin population of this invasive population with preliminary molecular analyses pointing to a Nova Scotia origin.

Given that this species was only formally described on the island within the last 5 years, and the oldest local account is within the last 50 years, we would assert this salamander arrived due to anthropogenic means. Our on-going molecular work (being prepared for submission) confirms local knowledge that they were brought to the island with agricultural goods from the mainland. Given the current widespread distribution of sighting that have been reported since the initial Baxter-Gilbert et al. (2022) report, it would suggest that populations are experiencing ‘booms and busts’ as within-island movement of the species creating thriving and failing populations, spatially and temporally (akin to the Opportunities Windows Hypothesis; see Johnstone 1986). As such we feel most comfortable using the term invasive when describing this Newfoundland salamander population as they were introduced due to human activity, survived, established a self-sustaining population, and have spread outward from the unknown introduction site to a series of different locations; thus, ticking all of the criteria from the Richardson et al. (2000) definition.

**Line 168: lower case p in** **plethodontid**

Thank you for noting this. We have corrected the capitalization of “plethodontid” to lowercase (see Line: 168).

**Line 192: should be Red-backed. Check throughout**

Thank you for catching this typo. We have checked throughout the document and updated Red-back to Red-backed (See Lines: 192, 262 339, 517).

**Lines 198-200: Did the diets of males and females differ? Juveniles and adults?**

The diets between males and females did not differ substantially. To compare the diets of males and females we used a permutational multivariate analysis of variance (PERMANOVA). We have added text to the manuscript to the effect (see Lines: 308 and 365-366). The diets between juveniles and adults differed slightly not in food types eaten but in size. Juveniles consumed slightly smaller prey, which aligns with their smaller mouth size. We did not feel the need add the additional analysis into our manuscript.

**Line 219: There is no way to measure small prey items like mites and springtails with calipers. Is this a mistake?**

We can understand that this could appear unlikely, but with the right approach we assert it can be done with a high degree of confidence. Very small prey items such as mites and springtails were measured with digital calipers looking through the dissecting microscope. The smallest prey item had a width of 0.16 mm. Our calipers, however, have an accuracy of 0.0127 mm and can measure from 0 to 152.4 cm. By placing them under the dissection scope we could ensure we were positioning the calipers correctly. We have added text to the manuscript to this effect (see Lines: 221-222).

**Line 252: salamanders plural**

Thank you for catching this typo. We have changed ‘salamander’ to ‘salamanders’ (see Line: 252).

**Lines 256-259: I do not think that this approach is valid because it likely makes the diversity indices incomparable. Please explain exactly how the index was calculated for your own data and for the data extracted from the literature. For example, you could (and should), at least for your own data, calculate a diversity index for each individual animal’s stomach contents. This of course allows you to compare means statistically. Obviously, this isn’t doable for most of the published literature without having the raw data so how did you deal with it? You need to explain exactly what you did because some papers report mean diversity based on calculations of diversity per individual (Anthony et al. 2008 and Hantak et al. 2020 and Stuczka et al 2016) other papers do this differently by providing only an index for a sample.**

**I did not go to each of the 14 papers used in this study but there were issues with the ones I did examine which makes me skeptical that the others were handled properly.**

**For example, it is unclear how the authors arrived at H’ = 1.96 for the Anthony et al data. That paper does not provide raw data, and the mean H’ for the striped morph = 2.71 and unstriped = 2.39.**

**The supplemental data in Hantak et al provides a list of prey species and total numbers of each of those species/morphospecies in the study. Fig. 3 in that paper reports 8 indices for 6 localities and 2 morphs (in two seasons if the supplemental are included). Diversity indices could either be calculated from the list of prey, or extracted from Fig. 3 + or - the supplemental fig. The results would differ depending on which approach was used. So how was H’ derived in S2 for this paper?**

**The sample size reported in Table S2 for the Raimondo et al 2003 paper is not correct. It should be 56 not 263. Additionally, this paper only examined the 7 most common prey categories which is inappropriate to use for calculating H’, evenness and richness.**

**Also, it is problematic to include data with unknown sample size and those natural history notes with n=1. If you look at the relationship between sample size and H’ you find a positive one. Studies that examined more salamander stomach contents generally have higher diversity scores. This is true if you exclude the unknown sample size, the n=1s, and fix the sample size for the Raimondo et al paper. Furthermore, the natural history notes should be excluded because they tend to be unrepresentative examples of the diet. For example, one of the used notes documents *cinereus* diet from a carnivorous plant in a bog so of course that individual’s gut was filled with flies.**

**Unfortunately, these problems make it difficult to trust the remaining diversity data and so the niche breadth comparison doesn’t seem valid, especially because diversity indices could have been calculated differently and even more importantly, diversity scores are dependent on sample size.**

We appreciate these points brought up by Reviewer 3. Firstly, the data used in our analysis was collected by reaching out to the authors individually, from the supplementary materials, or extracted from figures. For example, for both the Anthony et al., 2008 and Stuczka et al., 2016 publications we reached out directly to Carl Anthony and used his raw data excel files for analysis. The H’ for Hantak et al., 2020 was derived from the provided supplementary materials. Unfortunately, Hantak did not respond to our requests for data and did not have the data listed separately for each study location we were unable to parse apart the 6 separate study locations.

Secondly, thank you for catching the error with our data collection from the Raimondo et al., 2006 paper. We have made the revision suggested.

We have now removed all papers were there was only a single salamander or feeding event noted and those where a full data is not accessible either in the text or from communication with authors. This was an excellent suggestion, and we appreciate Reviewer 3 flagging it. This action has now decreased the number of publications in our analysis from 14 to 8. Importantly, this did not changed the diversity indexes by very much, nor the overall outcome (i.e., the invasive population still showed a higher prey diversity score and more evenness), but it did demonstrate that the change in feeding strategy may be associated with a latitudinal gradient (see Lines 296-299 for Results, as well as 441-452 and 467-489 for Discussion).

**Line 287: Table S3 is confusing. The columns %N and %I seem like they should be combined in a column with Y or N. As is, the columns are all either 100% or 0%**

Thank you for your comment. We have updated the column in Table S3 to include if the prey item is native Y or N.

**Line 306: “compared” is missing before “to conspecifics”**

Thank you for this suggestion. We have added “compared” in front of “to conspecifics” (see Line 306).

**Line 354 Table 1: Collembola are missing**

**To me it is strange that all comparisons differ statistically in this table. What is the biological relevance of a difference of for example of 0.005% versus 0. What are the expected values for these tests and how are you interpreting these results?**

**Also from the table it appears as though species richness is greater in the native range but of course the sample size collectively is much bigger for the combined literature and you likely cannot report a mean richness for the native data.**

We thank Reviewer 3 for raising these important points regarding the interpretation of Table 1. We have triple-checked our data and updated the table accordingly, adding the missing data on Collembola into it. The χ² test was used to compare the proportional composition of prey categories between the native and invasive ranges, with the native-range diet serving as the expected distribution. Given the large total sample sizes derived from the literature, even small proportional differences between ranges can result in statistically significant χ² values. Overall, it is hard to know the biological relevance of a difference between 0.005% and 0, nevertheless it is a true representation of our data. As you previously noted above, all too often papers will only discuss the “dominant-prey groups” which then makes later comparison with that data difficult (as we have experienced here). As such, we feel it is appropriate to show all of the groups, their comparisons, and the statistical outcomes.

Regarding mean species richness, it is larger in the mainland, native range. As one would expect as this would cover a much larger diversity of prey communities. We feel it is still useful to report it, as the ideas was to compare the island diets to that of a generalised mean native value. That said, we made a point to contextualise this in the manuscript (see Lines: 492-517).

**Line 357 Table 2: I think if you carefully look at the literature on diets from the native range you will see that by number mites and Collembola make up the majority of the diet which mirrors your findings, except interestingly you found many beetles. Were those beetles larvae or adults?**

**Another interesting finding is that you found quite a few centipedes in your stomachs which by volume is significant and unique compared to the native range diets. Isopods are a common prey item in the native range as well. Check out the importance values in the literature that you used to extract diet data. Most of those are exotic species of isopods.**

We thank Reviewer 3 for these thoughtful observations and interpretations of the dietary patterns presented in Table 2. With respect to beetles, we can confirm that a large proportion of the beetles identified in our stomach content samples were adults rather than larvae. Furthermore, we agree that mites and collembolans constitute a substantial proportion of the diet in the native range by number, consistent with patterns reported in the literature and with our own findings. We also acknowledge the reviewer’s observation that centipedes and isopods represent notable prey items in our invasive population, particularly given their volumetric contribution and this likely has contributed to the outcomes we are presenting herein.

We appreciate this point brought up by Reviewer 3 on invasive prey items being in the native salamanders’ diets. Unfortunately, our field work was only completed on the invasive population. Our literature review compiled the general diet of *Plethodon cinereus* in their native range, covered 8 studies, and 18 populations, however the degree of taxonomic resolution for most of these studies was only as detailed to the Order or Family level, and often the authors did not distinguish between invasive/native prey. In general, most of the studies that were used in the literature analysis did not use specific enough levels of prey identification to identify if the prey was an invasive/native species. Therefore, we were unable to categorise and back-calculate the native or invasive status of each prey items from the literature review. One study from which we compiled data mentioned invasive prey items (see Scott et al, 2024). However, this study only examined earthworms as invasive prey items and did not consider that other prey items may have been invasive as well. We very much agree that such an examination would allow for more in-depth examinations of the diet – yielding strong data for comparison between invasive and native populations. It is our hope that our manuscript will prompt more detailed natural history data collection, allowing for the comparison the Reviewer has suggested to be done in the future.

**Mesostigmata appears in Table 2 twice**

We thank the reviewer for noting the duplication of Mesostigmata in Table 2. These rows have been combined and the table values corrected accordingly.

**Line 392: Table 3 does not give any additional data that are not in the text so I would get rid of it even if you can argue the validity of the info in Table S2.**

We have removed Table 3 from the manuscript.

**Lines 441-449: I think this paragraph is overwritten and too strongly worded. Without data on diet preferences which would require samples of the available prey you cannot say that the non-native population has shifted its dietary niche to non-native prey. BY number your samples still have mostly mites and springtails. In the native range this species is truly a generalist predator and for the most part they prey on whatever is in abundance (native or non-native prey) when foraging conditions are less than ideal (warm or dry). When conditions are cool and wet foraging occurs more broadly, and salamanders selectively consume prey that are soft bodied and pass through the gut quickly (i.e. less chitinous). We do know that *cinereus* can and do avoid certain available prey and select certain other prey (see Paluh et al 2015 which examines selective foraging on ants by striped and unstriped morphs of cinereus). Maybe *cinereus* in Newfoundland are selectively foraging on larger, chitinous prey that happen to be exotic, or maybe those are just the prey that were most available and offered the biggest for there buck when you sampled, but without knowing what prey taxa are available it would be difficult to say and certainly. you cannot claim a shift in diet from the native range without a clearer comparison to data that were collected and analyzed the same way. So minimally the writing must be toned down.**

This is an excellent point, and we appreciate Reviewer 3’s insight. We have not toned down the language that was suggestive of a shift in feeding behaviour from mainland to invasive populations. We agree that prey preference is a missing aspect of our understand of the dynamics of this populations feeding, and future studies intend to follow up with this work once our molecular work is done to pinpoint the original source populations. In the meantime, we have included statement noting the missing prey preference information’s (see Lines 544 - 548).

To soften the language of the first Discussion paragraph the text now reads: “*Our study provides evidence that the population of Eastern Red-backed Salamanders in Newfoundland may have undergone dietary shifts during establishment and also exploits pre-existing invasive prey communities. Both of these factors may have increased their ability to create niche opportunities and bolster their invasion success. We found that the invasive salamander population had a more diverse and even diet, when compared to native populations, however this could be either a product of an invasion-derived shift in diet or an extralimital continuation of the native range cline for more diverse diets at higher latitudes. Nevertheless, despite an increase in dietary diversity and evenness within the invasive population, overall dietary composition between the native and invasive range populations were not significantly different. Proportional differences in individual prey groups were detected, however, suggesting that while the general structure of the diet remains broadly similar, the relative importance of specific prey taxa differs between native and invasive range populations. This pattern partially aligns with predictions of the Niche Breadth Invasion Success Hypothesis [23]. We also found that the invasive salamander population feeds heavily on invasive prey items, both in abundance and biomass, supporting our other prediction and providing strong support for the Invasion Meltdown Hypothesis [32].*” (see Lines 441-456).

**Lines 478-484: I don’t think you can say that differences in the diets reported in this study represent real differences. This would be an assumption if we knew that the available prey communities were relatively the same in both native and non-native ranges, but we do not have that information. Richness was greater in the native range (Table 1) and the diversity indices are flawed so the statements in this part of the discussion are not supported by the data. Additionally, we know from papers published by both BM Walton and RG Jaeger that litter invertebrate prey populations fluctuate drastically by season primarily due to litter thickness and moisture. Such fluctuations result in seasonal differences in competition for prey. Competition weakens in late spring and fall when resources, and good foraging conditions, are abundant. In the summer and early fall competition for limited or lower quality resources resumes resulting in partitioning of the prey base in ways that territorial residents monopolize the best foraging areas while floaters are only able to acquire lower quality prey (those that are heavily armored and pass through the gut more slowly). This is another reason that comparing your data to combined data from other studies is problematic. I am sure data were collected from all seasons in the native range dataset whereas these data were collected in May and June.**

It is very clear that Reviewer 3 has extensive expertise with the ecology of this species, and we continually appreciate their insights. Notably, regarding the detailed specificity of changes in the prey composition and availability for this species. If we were intending on conducting a site-to-site comparison, such information of variation and comparability based on seasonality, elevation, forest floor dynamics, and so on would be highly pertinent. And as we are intending to do this next (once the molecular work is put to bed) we really value their insights on how to ensure the details are account for with respect to specific site-to-site comparisons. Thank you!

Regarding our current study, however, we are endeavouring to contrast the diet of the invasive salamander of Newfoundland to a “typical” diet of an Eastern Red-backed Salamander that is generalised from across the native range. Here we do not have the ability of controlling for factors such as time of year, elevations, leaf litter depth, and so on. And in all honestly due to the expansive difference in geography, it would not actually matter. What is happening on the forest floor in Newfoundland during May/June is very different to that from near the Great Lakes or the Appalachians at the same time. Furthermore, the fact of the matter is, despite a long history of studying this species, which has provided a wealth of information, we still lack an appropriately robust dataset of the true diet of this salamander across the native range that would allow us to be picky with site specific minutia. What we have found during this work is that very few studies report the entire dietary breadth (as Reviewer 3 rightly notes; see above), even fewer provide lists of dietary composition that are taxonomically specific (e.g., to species- or genus-level; as we detail in this manuscript). In short, after undertaking a systematic literature review, we only found 14 studies from which to derive a mean dietary description (less so now, given the trimming this review has rightly identified).

Overall, what we are presenting here is a series of evidence that suggests that compared to a generalised Eastern Red-backed Salamander diet, the invasive salamanders of Newfoundland are appearing to feed more broadly (i.e., a generalist predator that is even more generalised) and feeding on a high proportion of invasive prey items – both of which should serve to minimise the risk of competitive exclusion with native taxa, and thus bolster invasion success.

**Bibliography on supplement – The Hughes citation was in issue 3, not 2**

Thank you for catching this typo. This publication has been removed during the course of this revision.

**My review is not meant to be negative. I think there are good diet data here on a population of red-backed salamanders that is introduced to Newfoundland which we know little about. My recommendation would be to focus on the non-native data and discuss them in the context of what is known about foraging tactics in this species.**

We appreciate your insight for this review, and understand your suggestions are made to be helpful. Given that this will be the second only publication to data on this invasive population of Eastern Red-backed Salamander in Newfoundland, we agree the value to better contextualise their data is clear. That said, we also feel it is important to not change the purpose of our study, nor re-jig our hypotheses after the data has come in (i.e., avoiding the issues associated with post hoc hypothesis or HARKing). We collected this data and analysed it with the intention of testing these hypotheses, and we feel it is ethical and appropriate to continue to do so. Furthermore, we also feel that with the data we have, we can reasonably make these comparison (especially given the data cleaning that was recommended; which we very much appreciate).

**Jaeger 1990 and citation therein would be a great place to start. You may be able to add a quick experiment to your diet data to test some of the hypotheses laid out in this paper.**

**Jaeger, Robert G. "Territorial salamanders evaluate size and chitinous content of arthropod prey." In *Behavioural mechanisms of food selection*, pp. 111-126. Berlin, Heidelberg: Springer Berlin Heidelberg, 1990.**

**Another helpful paper**

**Daniel J. Paluh, Cameron Eddy, Kaloyan Ivanov, Cari-Ann M. Hickerson, and Carl D. Anthony "Selective Foraging on Ants by a Terrestrial Polymorphic Salamander," *The American Midland Naturalist* 174(2), 265-277, (1 October 2015).** [**https://doi.org/10.1674/0003-0031-174.2.265**](https://doi.org/10.1674/0003-0031-174.2.265)

Thank you so much for taking the time to thoroughly review our manuscript, and for providing additional reading on this topic.

**Alternatively, if you choose to keep the focus on the extracted data from the literature, I would make certain that the data are comparable which likely means excluding some of the papers and acquiring raw data from authors to ensure that the analyses and calculations are the same and done correctly. This would include ensuring similar sample sizes from native and non-native ranges with data collected at the same time of year and at similar elevation.**

We have opted to take this approach and have endeavoured to ensure our data is comparable. When possible, we have used raw data from authors (when it is not present in the published manuscript or supplementary material) and removed those that could not be analysed do to biases or missing data. Give that the invasive population has only 129 stomach samples for diet, balancing the samples sizes was not possible. Given our goal was to create a generalised mean measure of dietary diversity. Similarly, time of year could not be control for due to the relative paucity of high-quality reporting on the entire diet of this species across the native range. Nevertheless, the suggestions made by the reviewer have greatly improved our manuscript, and we very much appreciate it!
