# Supplementary material for: In the belly of the beast: How dietary changes and preexisting invasive prey may have promoted the success of a novel invasive amphibian
Source: PLoS One. 2026 Jul 10;21(7):e0352970. doi: 10.1371/journal.pone.0352970 (PMC13354068; doi:10.1371/journal.pone.0352970)
Supplement: Peer review history

# Peer review: Decision Letter 0

**DOI:** 10.1371/journal.pone.0352970.r001

**Authors:** Alexandre Ribeiro da Silva

29 Jul 2025

PONE-D-25-22281

In the Belly of the Beast: How Dietary Changes and Preexisting Invasive Prey may have Promoted the Success of a Novel Invasive Amphibian

PLOS ONE

Dear Dr. Baxter-Gilbert,

Thank you for submitting your manuscript to PLOS ONE. After careful consideration, we feel that it has merit but does not fully meet PLOS ONE’s publication criteria as it currently stands. Therefore, we invite you to submit a revised version of the manuscript that addresses the points raised during the review process.

Please submit your revised manuscript by Sep 12 2025 11:59PM. If you will need more time than this to complete your revisions, please reply to this message or contact the journal office at plosone@plos.org. When you’re ready to submit your revision, log on to https://www.editorialmanager.com/pone/ and select the 'Submissions Needing Revision' folder to locate your manuscript file.

Please include the following items when submitting your revised manuscript:

- A rebuttal letter that responds to each point raised by the academic editor and reviewer(s). You should upload this letter as a separate file labeled 'Response to Reviewers'.
- A marked-up copy of your manuscript that highlights changes made to the original version. You should upload this as a separate file labeled 'Revised Manuscript with Track Changes'.
- An unmarked version of your revised paper without tracked changes. You should upload this as a separate file labeled 'Manuscript'.

-->If you would like to make changes to your financial disclosure, please include your updated statement in your cover letter. Guidelines for resubmitting your figure files are available below the reviewer comments at the end of this letter.

If applicable, we recommend that you deposit your laboratory protocols in protocols.io to enhance the reproducibility of your results. Protocols.io assigns your protocol its own identifier (DOI) so that it can be cited independently in the future. For instructions see: https://journals.plos.org/plosone/s/submission-guidelines#loc-laboratory-protocols. Additionally, PLOS ONE offers an option for publishing peer-reviewed Lab Protocol articles, which describe protocols hosted on protocols.io. Read more information on sharing protocols at https://plos.org/protocols?utm_medium=editorial-email&utm_source=authorletters&utm_campaign=protocols.

We look forward to receiving your revised manuscript.

Kind regards,

Alexandre Ribeiro da Silva

Academic Editor

PLOS ONE

Journal Requirements:

When submitting your revision, we need you to address these additional requirements.

1. Please ensure that your manuscript meets PLOS ONE's style requirements, including those for file naming. The PLOS ONE style templates can be found at

https://journals.plos.org/plosone/s/file?id=wjVg/PLOSOne_formatting_sample_main_body.pdf and

https://journals.plos.org/plosone/s/file?id=ba62/PLOSOne_formatting_sample_title_authors_affiliations.pdf

2. Thank you for stating the following financial disclosure:

1)

Author's Initials: M. J. W.

Award Name: J. E. A. Crake Foundation Grant

Funder Names: Mount Allison University

2)

Author's Initials: J. B-G.

Award Name: Part-time Research Stipend

Funder Names: Mount Allison University

3)

Author's Initials: J. L. R.

Award Name: Discovery Grant

Funder Names: Natural Sciences and Engineering Research Council of Canada (NSERC)

Please state what role the funders took in the study. If the funders had no role, please state: "The funders had no role in study design, data collection and analysis, decision to publish, or preparation of the manuscript."

If this statement is not correct you must amend it as needed.

Please include this amended Role of Funder statement in your cover letter; we will change the online submission form on your behalf.

3. We note that Figure 2 in your submission contain map images which may be copyrighted. All PLOS content is published under the Creative Commons Attribution License (CC BY 4.0), which means that the manuscript, images, and Supporting Information files will be freely available online, and any third party is permitted to access, download, copy, distribute, and use these materials in any way, even commercially, with proper attribution. For these reasons, we cannot publish previously copyrighted maps or satellite images created using proprietary data, such as Google software (Google Maps, Street View, and Earth). For more information, see our copyright guidelines: http://journals.plos.org/plosone/s/licenses-and-copyright.

We require you to either (a) present written permission from the copyright holder to publish these figures specifically under the CC BY 4.0 license, or (b) remove the figures from your submission:

a. You may seek permission from the original copyright holder of Figure 2 to publish the content specifically under the CC BY 4.0 license.

We recommend that you contact the original copyright holder with the Content Permission Form (http://journals.plos.org/plosone/s/file?id=7c09/content-permission-form.pdf) and the following text:

“I request permission for the open-access journal PLOS ONE to publish XXX under the Creative Commons Attribution License (CCAL) CC BY 4.0 (http://creativecommons.org/licenses/by/4.0/). Please be aware that this license allows unrestricted use and distribution, even commercially, by third parties. Please reply and provide explicit written permission to publish XXX under a CC BY license and complete the attached form.”

Please upload the completed Content Permission Form or other proof of granted permissions as an ""Other"" file with your submission.

In the figure caption of the copyrighted figure, please include the following text: “Reprinted from [ref] under a CC BY license, with permission from [name of publisher], original copyright [original copyright year].”

b. If you are unable to obtain permission from the original copyright holder to publish these figures under the CC BY 4.0 license or if the copyright holder’s requirements are incompatible with the CC BY 4.0 license, please either i) remove the figure or ii) supply a replacement figure that complies with the CC BY 4.0 license. Please check copyright information on all replacement figures and update the figure caption with source information. If applicable, please specify in the figure caption text when a figure is similar but not identical to the original image and is therefore for illustrative purposes only.

The following resources for replacing copyrighted map figures may be helpful:

USGS National Map Viewer (public domain): http://viewer.nationalmap.gov/viewer/

The Gateway to Astronaut Photography of Earth (public domain): http://eol.jsc.nasa.gov/sseop/clickmap/

Maps at the CIA (public domain): https://www.cia.gov/library/publications/the-world-factbook/index.html and https://www.cia.gov/library/publications/cia-maps-publications/index.html

NASA Earth Observatory (public domain): http://earthobservatory.nasa.gov/

Landsat: http://landsat.visibleearth.nasa.gov/

USGS EROS (Earth Resources Observatory and Science (EROS) Center) (public domain): http://eros.usgs.gov/#

Natural Earth (public domain): http://www.naturalearthdata.com/

4. Please include captions for your Supporting Information files at the end of your manuscript, and update any in-text citations to match accordingly. Please see our Supporting Information guidelines for more information: http://journals.plos.org/plosone/s/supporting-information.

5. Please remove all personal information, ensure that the data shared are in accordance with participant consent, and re-upload a fully anonymized data set.

Note: spreadsheet columns with personal information must be removed and not hidden as all hidden columns will appear in the published file.

Additional guidance on preparing raw data for publication can be found in our Data Policy (https://journals.plos.org/plosone/s/data-availability#loc-human-research-participant-data-and-other-sensitive-data) and in the following article: http://www.bmj.com/content/340/bmj.c181.long.

6. If the reviewer comments include a recommendation to cite specific previously published works, please review and evaluate these publications to determine whether they are relevant and should be cited. There is no requirement to cite these works unless the editor has indicated otherwise.

7. Please review your reference list to ensure that it is complete and correct. If you have cited papers that have been retracted, please include the rationale for doing so in the manuscript text, or remove these references and replace them with relevant current references. Any changes to the reference list should be mentioned in the rebuttal letter that accompanies your revised manuscript. If you need to cite a retracted article, indicate the article’s retracted status in the References list and also include a citation and full reference for the retraction notice.

Additional Editor Comments:

Dear authors,

Thanks for your submission to PLOS One.

I've carefully read the manuscript, and I agree with the reviewers that it needs some revision.

I've enjoyed reading it, since I work with biological invasion as well, albeit in marine environments.

I have one minor issue that I think could be addressed.

I believe the authors should include a paragraph stating the limitations of the study.

I say this in the sense that:

First: Diet is often linked to food availability. This was not measured.

The authors briefly stated at the end of the discussion that the availability of non-native species was not measured.

Thus, we cannot say for sure that it contributes to the establishment of Plethodon.

But one thing is certain: this species has plasticity regarding its feeding niche, which probably contributed to its status as an invasive species.

Second: I'd like to congratulate the authors on the extensive literature review.

However, some of the manuscripts date back to the 1980s and 1990s. That said, the amphibians' diet could have changed over time due to the potential introduction of new non-native prey. It is something to think about.

Therefore, I suggest adding a paragraph discussing these limitations.

Overall, I've found the manuscript really interesting and enjoyable.

Best regards,

Alex

[Note: HTML markup is below. Please do not edit.]

Reviewers' comments:

Reviewer's Responses to Questions

**Comments to the Author**

1. Is the manuscript technically sound, and do the data support the conclusions?

The manuscript must describe a technically sound piece of scientific research with data that supports the conclusions. Experiments must have been conducted rigorously, with appropriate controls, replication, and sample sizes. The conclusions must be drawn appropriately based on the data presented.

Reviewer #1: Yes

Reviewer #2: Yes

**********

2. Has the statistical analysis been performed appropriately and rigorously?

Reviewer #1: Yes

Reviewer #2: Yes

**********

3. Have the authors made all data underlying the findings in their manuscript fully available?

The PLOS Data policy requires authors to make all data underlying the findings described in their manuscript fully available without restriction, with rare exception (please refer to the Data Availability Statement in the manuscript PDF file). The data should be provided as part of the manuscript or its supporting information, or deposited to a public repository. For example, in addition to summary statistics, the data points behind means, medians and variance measures should be available. If there are restrictions on publicly sharing data—e.g. participant privacy or use of data from a third party—those must be specified.

Reviewer #1: Yes

Reviewer #2: Yes

**********

4. Is the manuscript presented in an intelligible fashion and written in standard English?

PLOS ONE does not copyedit accepted manuscripts, so the language in submitted articles must be clear, correct, and unambiguous. Any typographical or grammatical errors should be corrected at revision, so please note any specific errors here.

Reviewer #1: Yes

Reviewer #2: Yes

**********

5. Review Comments to the Author

Please use the space provided to explain your answers to the questions above. You may also include additional comments for the author, including concerns about dual publication, research ethics, or publication ethics. (Please upload your review as an attachment if it exceeds 20,000 characters)

Reviewer #1: Dear Authors,

First and foremost, congratulations on the work and effort invested in its realization and in advancing scientific knowledge regarding these ecological relationships. Moving on to the review, I have provided suggestions for reflection and alteration, applicable to either this study or future investigations.

Minor issues:

(1) The results can inform invasive species management strategies. If the predatory species is predominantly feeding on other invasive species in terms of volume, this could be considered an ecosystem service (if the objective is to control these invasive prey). However, if the priority is to protect native species that are also consumed (even if in smaller volume), removal of the invasive predatory species might be necessary to reduce pressure on native fauna (Simberloff, 2003). Could the study propose management that considers the balance between the direct and indirect negative impacts of the invasive species across all its trophic interactions?

(2) Results – When analyzing the consumed volume, there is a highly significant difference, with invasive prey contributing a much larger proportion of the total diet volume (~67.6%) compared to native prey. What explains this?

(3) Results – I perceive a statistically very significant difference in the volumes of prey consumed, depending on their status (Native vs. Invasive), by calculating the proportion of the volume. This contrasts with the analysis of percentage by individual, which was not significant. What accounts for this contrast? Please discuss further.

(4) Analysis – Indicator Species Analysis (IndVal) – Identify which prey items are significant indicators of native or invasive populations, or of certain geographical regions. This could reveal specializations or niche shifts.

Optional:

(1) Methods – Although the percentage volume of invasive vs. native prey per individual specimen was not significantly different (p = 0.075), the analysis of aggregated total volume reveals that the consumption of invasive prey has a substantially greater contribution to the total ingested biomass. In summary, despite the absence of clear geographical patterns, the diet of the studied species is strongly influenced by invasive prey in terms of total consumed biomass, which can have significant ecological implications for trophic dynamics and the success of the invasive population. The analyses performed provided important insights but also raised questions that could be further explored in this work and/or for future investigations, given the existing limitations of methods and collected data. Consider the following points if deemed necessary and coherent.

(2) Analysis – Multivariate Models for Diet Diversity GLM(M) Multiple – Instead of modeling Shannon diversity with just Lat or Log separately, combine them into a single model: Shannon_Index ~ Lat + Log. Add other environmental variables or population/specimen characteristics that might influence the diet (e.g., individual size, predator sex, habitat type, local temperature, precipitation, altitude). This would help understand the relative contribution of each factor and if they interact. – If this article aims to answer this, clearly state the limitation if it is not achieved.

(3) Methods – Both models show a positive trend for their respective predictors (Lat and Log). Neither predictor achieves statistical significance at the 0.05 level. The model with Log (g2) has a slightly lower AIC, suggesting it explains slightly more of the variation in Shannon diversity than the model with Lat, although the difference is minimal and significance is still marginal. This indicates that neither latitude nor longitude, individually, are strong predictors of diet diversity in these univariate analyses, although there is a suggestion that diversity may increase at higher latitudes and longitudes. It would be interesting to explore a model that included both variables or others, or consider the interaction between them, to see if a combination better explains the variation in diet diversity. Subsequently, the results suggest that both latitude and longitude have a limited and statistically non-significant influence (at the standard confidence level) on individual diet diversity. Although there is a trend of increasing diversity with higher latitudes and longitudes, this is not robust enough to be considered a clear and predictive relationship based on this dataset. This might indicate that other factors not included in the models (climatic, environmental, biological) or the complexity of geospatial interactions may be more important, or that the sample size is insufficient to detect more subtle effects (just make this clear in the text, limitations are fine). This suggests a trend of increasing diet diversity with longitude, but the evidence is not strong enough to reject the null hypothesis (that there is no relationship) – A question to be posed for long-term future study investigations.

Reviewer #2: Review for:

PONE-D-25-22281: In the Belly of the Beast: How Dietary Changes and Preexisting Invasive Prey may have Promoted the Success of a Novel Invasive Amphibian

Synopsis:

The authors compared diets of invasive and native range salamanders to determine whether invaders had a wider dietary niche. They found that invasive populations had significantly wider dietary niche which included higher rates of invasive prey, potentially reflecting a ‘invasional meltdown’ scenario that had enabled the salamanders’ expansion into a novel island ecosystem.

General comments:

This paper provided an excellent overview of multiple invasion theories and prior literature. It was well set up to neatly test the authors questions. The methods and stats are all sound and I only had a few issues to raise in addition to some line by line comments. I believe once rectified, the paper will be suitable for publication.

1. The introduction (whilst very informative) is somewhat clunky in places. There are some line by line comments below, but I found that overall, many of the sentences were too long and thus felt like ‘information overload’. Please go over the introduction and break up some of the sentences, plus remove word ‘double ups’ in the same or close by sentences.

2. The authors did not make direct comparisons between the invasive and native range population in terms of how much invasive prey they consumed. As far as I can tell – only the invasive population’s diet was examined in this way. But that’s an important part of the interpretation. If native range population eat equal amounts of invasive prey then that changes the story. Please include more results and subsequent discussion of this aspect directly.

3. A little more explanation is required regarding the invasive population status – how are the authors certain that these populations are invasive? Is a delay in reporting animals on the island merely because there were less scrutiny of the biodiversity previously? Do island inhabitants remember the invasion arriving etc. It seems unusual that these animals wouldn’t be found on close by islands, but perhaps not for someone that knows the system better.

Line by line (note: I used the line numbers of written text on the pdf proof)

37: ‘bolstered’?

49: too close to use ‘across the globe’ again.

58/59: clunky use of ‘increase’ twice – reword.

60/61: double use of expanding in one sentence

73: reword ‘ their introductions into Mauritius and Tanzania both failed’.

78 – 86: great overview!

91/92: sentence to convoluted – divide.

152: compared ‘it’? be careful with your wording – I would change the whole sentence to be clearer: e.g. To do investigate this, we compared the diets of invasive vs. non-invasive populations of salamanders. We analysed stomach contents form the invasive populations and did systematic literature reviews to characterise the diet of the species in its natural range.

177: do you know how or why they got there?

195: how do you sex them?

221: could some species be missed in the literature because they are digested prior to analysis?

239: has this species name stayed consistent since 1967?

282: ‘how frequently’?

328 and 338, 387 and 388, 392 etc.: include common names also where possible (e.g. ‘mites’, ‘beetles’, ‘nematodes’) as it keeps the text more engaging.

405: was there any invasive prey in the native range diets? I can’t see that mentioned anywhere but that seems important.

447: interesting!

507: I would have thought this would be relatively easy to do from literature already?

**********

6. PLOS authors have the option to publish the peer review history of their article (what does this mean?). If published, this will include your full peer review and any attached files.

If you choose “no”, your identity will remain anonymous but your review may still be made public.

**Do you want your identity to be public for this peer review?**  For information about this choice, including consent withdrawal, please see our Privacy Policy.

Reviewer #1: No

Reviewer #2: No

**********

[NOTE: If reviewer comments were submitted as an attachment file, they will be attached to this email and accessible via the submission site. Please log into your account, locate the manuscript record, and check for the action link "View Attachments". If this link does not appear, there are no attachment files.]

While revising your submission, please upload your figure files to the Preflight Analysis and Conversion Engine (PACE) digital diagnostic tool, https://pacev2.apexcovantage.com/. PACE helps ensure that figures meet PLOS requirements. To use PACE, you must first register as a user. Registration is free. Then, login and navigate to the UPLOAD tab, where you will find detailed instructions on how to use the tool. If you encounter any issues or have any questions when using PACE, please email PLOS at figures@plos.org. Please note that Supporting Information files do not need this step.

# Author comment: Author response to Decision Letter 1

**DOI:** 10.1371/journal.pone.0352970.r002

17 Oct 2025

Please see the attached file "Response to Reviewers" for a detailed set of responses for the associate editor's and both reviewer's comments

Attachment Submitted filename: Response to Reviewers_Williams et al_final.docx

# Peer review: Decision Letter 1

**DOI:** 10.1371/journal.pone.0352970.r003

**Authors:** Alexandre Ribeiro da Silva

8 Dec 2025

PONE-D-25-22281R1

In the Belly of the Beast: How Dietary Changes and Preexisting Invasive Prey may have Promoted the Success of a Novel Invasive Amphibian

PLOS One

Dear Dr. Baxter-Gilbert,

Thank you for submitting your manuscript to PLOS ONE. After careful consideration, we feel that it has merit but does not fully meet PLOS ONE’s publication criteria as it currently stands. Therefore, we invite you to submit a revised version of the manuscript that addresses the points raised during the review process.

==============================

**Dear Authors,**

I am writing to inform you that I am recommending your manuscript for **minor revisions** .

Please note that one of the reviewers from the first round was unavailable to continue, and therefore a **new reviewer**  was invited. As a result, additional comments have been raised in this round.

I understand that this process can be time-consuming and, at times, demanding. However, these steps are essential to ensure the **scientific rigor, clarity, and overall quality**  of the manuscript.

Thank you for your continued cooperation. Please feel free to reach out if clarification is needed during the revision process.

==============================

Please submit your revised manuscript by Jan 22 2026 11:59PM. If you will need more time than this to complete your revisions, please reply to this message or contact the journal office at plosone@plos.org. When you're ready to submit your revision, log on to https://www.editorialmanager.com/pone/ and select the 'Submissions Needing Revision' folder to locate your manuscript file.

Please include the following items when submitting your revised manuscript:

- A rebuttal letter that responds to each point raised by the academic editor and reviewer(s). You should upload this letter as a separate file labeled 'Response to Reviewers'.
- A marked-up copy of your manuscript that highlights changes made to the original version. You should upload this as a separate file labeled 'Revised Manuscript with Track Changes'.
- An unmarked version of your revised paper without tracked changes. You should upload this as a separate file labeled 'Manuscript'.

-->If you would like to make changes to your financial disclosure, please include your updated statement in your cover letter. Guidelines for resubmitting your figure files are available below the reviewer comments at the end of this letter.

If applicable, we recommend that you deposit your laboratory protocols in protocols.io to enhance the reproducibility of your results. Protocols.io assigns your protocol its own identifier (DOI) so that it can be cited independently in the future. For instructions see: https://journals.plos.org/plosone/s/submission-guidelines#loc-laboratory-protocols. Additionally, PLOS ONE offers an option for publishing peer-reviewed Lab Protocol articles, which describe protocols hosted on protocols.io. Read more information on sharing protocols at https://plos.org/protocols?utm_medium=editorial-email&utm_source=authorletters&utm_campaign=protocols.

We look forward to receiving your revised manuscript.

Kind regards,

Alexandre Ribeiro da Silva

Academic Editor

PLOS One

**Journal Requirements:**

1. If the reviewer comments include a recommendation to cite specific previously published works, please review and evaluate these publications to determine whether they are relevant and should be cited. There is no requirement to cite these works unless the editor has indicated otherwise.

2. Please review your reference list to ensure that it is complete and correct. If you have cited papers that have been retracted, please include the rationale for doing so in the manuscript text, or remove these references and replace them with relevant current references. Any changes to the reference list should be mentioned in the rebuttal letter that accompanies your revised manuscript. If you need to cite a retracted article, indicate the article’s retracted status in the References list and also include a citation and full reference for the retraction notice.

[Note: HTML markup is below. Please do not edit.]

Reviewers' comments:

Reviewer's Responses to Questions

**Comments to the Author**

1. If the authors have adequately addressed your comments raised in a previous round of review and you feel that this manuscript is now acceptable for publication, you may indicate that here to bypass the “Comments to the Author” section, enter your conflict of interest statement in the “Confidential to Editor” section, and submit your "Accept" recommendation.

Reviewer #1: All comments have been addressed

Reviewer #3: (No Response)

**********

2. Is the manuscript technically sound, and do the data support the conclusions?

The manuscript must describe a technically sound piece of scientific research with data that supports the conclusions. Experiments must have been conducted rigorously, with appropriate controls, replication, and sample sizes. The conclusions must be drawn appropriately based on the data presented.

Reviewer #1: Yes

Reviewer #3: No

**********

3. Has the statistical analysis been performed appropriately and rigorously?

Reviewer #1: Yes

Reviewer #3: I Don't Know

**********

4. Have the authors made all data underlying the findings in their manuscript fully available?

The PLOS Data policy requires authors to make all data underlying the findings described in their manuscript fully available without restriction, with rare exception (please refer to the Data Availability Statement in the manuscript PDF file). The data should be provided as part of the manuscript or its supporting information, or deposited to a public repository. For example, in addition to summary statistics, the data points behind means, medians and variance measures should be available. If there are restrictions on publicly sharing data—e.g. participant privacy or use of data from a third party—those must be specified.

Reviewer #1: Yes

Reviewer #3: Yes

**********

5. Is the manuscript presented in an intelligible fashion and written in standard English?

PLOS ONE does not copyedit accepted manuscripts, so the language in submitted articles must be clear, correct, and unambiguous. Any typographical or grammatical errors should be corrected at revision, so please note any specific errors here.

Reviewer #1: Yes

Reviewer #3: Yes

**********

6. Review Comments to the Author

Please use the space provided to explain your answers to the questions above. You may also include additional comments for the author, including concerns about dual publication, research ethics, or publication ethics. (Please upload your review as an attachment if it exceeds 20,000 characters)

**Reviewer #1:**  The answers provided were satisfactory. There are changes to be made, but overall, questions were answered and clarified.

**Reviewer #3:**  uploaded as a separate file

This paper reports the stomach contents of a robust sample of the population of Eastern Red-backed Salamanders on Newfoundland and attempts to make comparisons to the existing diet data for this species from its native range. The paper is well written but, in my view, needs to be recast in the context of optimal foraging and natural history of the species. The niche breadth analysis is not correct and the interpretation of the data is an overreach.

**********

7. PLOS authors have the option to publish the peer review history of their article (what does this mean?). If published, this will include your full peer review and any attached files.

If you choose “no”, your identity will remain anonymous but your review may still be made public.

**Do you want your identity to be public for this peer review?**  For information about this choice, including consent withdrawal, please see our Privacy Policy.

Reviewer #1: **Yes:**  Marcos Akira-Umeno

Reviewer #3: No

**********

[NOTE: If reviewer comments were submitted as an attachment file, they will be attached to this email and accessible via the submission site. Please log into your account, locate the manuscript record, and check for the action link "View Attachments". If this link does not appear, there are no attachment files.]

To ensure your figures meet our technical requirements, please review our figure guidelines: https://journals.plos.org/plosone/s/figures

You may also use PLOS’s free figure tool, NAAS, to help you prepare publication quality figures: https://journals.plos.org/plosone/s/figures#loc-tools-for-figure-preparation.

NAAS will assess whether your figures meet our technical requirements by comparing each figure against our figure specifications.

Attachment Submitted filename: Review-eng-mau-02.docx

Attachment Submitted filename: PONE-D-25-22281R1.docx

# Author comment: Author response to Decision Letter 2

**DOI:** 10.1371/journal.pone.0352970.r004

8 Jun 2026

Please see the attached file for the full response to reviewers

Attachment Submitted filename: Response to Reviewers v5_Williams et al.docx

# Peer review: Decision Letter 2

**DOI:** 10.1371/journal.pone.0352970.r005

**Authors:** Alexandre Ribeiro da Silva

18 Jun 2026

In the Belly of the Beast: How Dietary Changes and Preexisting Invasive Prey may have Promoted the Success of a Novel Invasive Amphibian

PONE-D-25-22281R2

Dear Dr. Baxter-Gilbert,

We’re pleased to inform you that your manuscript has been judged scientifically suitable for publication and will be formally accepted for publication once it meets all outstanding technical requirements.

Within one week, you’ll receive an e-mail detailing the required amendments. When these have been addressed, you’ll receive a formal acceptance letter and your manuscript will be scheduled for publication.

An invoice will be generated when your article is formally accepted. Please note, if your institution has a publishing partnership with PLOS and your article meets the relevant criteria, all or part of your publication costs will be covered. Please make sure your user information is up-to-date by logging into Editorial Manager at Editorial Manager® and clicking the ‘Update My Information' link at the top of the page. For questions related to billing, please contact billing support.

If your institution or institutions have a press office, please notify them about your upcoming paper to help maximize its impact. If they’ll be preparing press materials, please inform our press team as soon as possible -- no later than 48 hours after receiving the formal acceptance. Your manuscript will remain under strict press embargo until 2 pm Eastern Time on the date of publication. For more information, please contact onepress@plos.org.

Kind regards,

Alexandre Ribeiro da Silva

Academic Editor

PLOS One

Additional Editor Comments (optional):

Reviewers' comments:

Reviewer's Responses to Questions

**Comments to the Author**

1. If the authors have adequately addressed your comments raised in a previous round of review and you feel that this manuscript is now acceptable for publication, you may indicate that here to bypass the “Comments to the Author” section, enter your conflict of interest statement in the “Confidential to Editor” section, and submit your "Accept" recommendation.

Reviewer #1: All comments have been addressed

**********

2. Is the manuscript technically sound, and do the data support the conclusions?

The manuscript must describe a technically sound piece of scientific research with data that supports the conclusions. Experiments must have been conducted rigorously, with appropriate controls, replication, and sample sizes. The conclusions must be drawn appropriately based on the data presented.

Reviewer #1: Yes

**********

3. Has the statistical analysis been performed appropriately and rigorously?

Reviewer #1: Yes

**********

4. Have the authors made all data underlying the findings in their manuscript fully available?

The PLOS Data policy requires authors to make all data underlying the findings described in their manuscript fully available without restriction, with rare exception (please refer to the Data Availability Statement in the manuscript PDF file). The data should be provided as part of the manuscript or its supporting information, or deposited to a public repository. For example, in addition to summary statistics, the data points behind means, medians and variance measures should be available. If there are restrictions on publicly sharing data—e.g. participant privacy or use of data from a third party—those must be specified.

Reviewer #1: Yes

**********

5. Is the manuscript presented in an intelligible fashion and written in standard English?

PLOS ONE does not copyedit accepted manuscripts, so the language in submitted articles must be clear, correct, and unambiguous. Any typographical or grammatical errors should be corrected at revision, so please note any specific errors here.

Reviewer #1: Yes

**********

6. Review Comments to the Author

Please use the space provided to explain your answers to the questions above. You may also include additional comments for the author, including concerns about dual publication, research ethics, or publication ethics. (Please upload your review as an attachment if it exceeds 20,000 characters)

Reviewer #1: The authors have addressed the points I raised; I am satisfied with the corrections and the improvements made to the paper.

**********

7. PLOS authors have the option to publish the peer review history of their article (what does this mean?). If published, this will include your full peer review and any attached files.

If you choose “no”, your identity will remain anonymous but your review may still be made public.

**Do you want your identity to be public for this peer review?**  For information about this choice, including consent withdrawal, please see our Privacy Policy.

Reviewer #1: **Yes:**  Marcos Akira-Umeno

**********

# Editor report: Acceptance letter

**DOI:** 10.1371/journal.pone.0352970.r006

**Authors:** Alexandre Ribeiro da Silva

PONE-D-25-22281R2

PLOS One

Dear Dr. Baxter-Gilbert,

I'm pleased to inform you that your manuscript has been deemed suitable for publication in PLOS One. Congratulations! Your manuscript is now being handed over to our production team.

At this stage, our production department will prepare your paper for publication. This includes ensuring the following:

* All references, tables, and figures are properly cited

* All relevant supporting information is included in the manuscript submission,

* There are no issues that prevent the paper from being properly typeset

You will receive further instructions from the production team, including instructions on how to review your proof when it is ready. Please keep in mind that we are working through a large volume of accepted articles, so please give us a few days to review your paper and let you know the next and final steps.

Lastly, if your institution or institutions have a press office, please let them know about your upcoming paper now to help maximize its impact. If they'll be preparing press materials, please inform our press team within the next 48 hours. Your manuscript will remain under strict press embargo until 2 pm Eastern Time on the date of publication. For more information, please contact onepress@plos.org.

You will receive an invoice from PLOS for your publication fee after your manuscript has reached the completed accept phase. If you receive an email requesting payment before acceptance or for any other service, this may be a phishing scheme. Learn how to identify phishing emails and protect your accounts at https://explore.plos.org/phishing.

If we can help with anything else, please email us at customercare@plos.org.

Thank you for submitting your work to PLOS ONE and supporting open access.

Kind regards,

PLOS ONE Editorial Office Staff

on behalf of

Dr. Alexandre Ribeiro da Silva

Academic Editor

PLOS One
